# Multistrategy-Boosted Carnivorous Plant Algorithm: Performance Analysis and Application in Engineering Designs

**DOI:** 10.3390/biomimetics8020162

**Published:** 2023-04-17

**Authors:** Min Peng, Wenlong Jing, Jianwei Yang, Gang Hu

**Affiliations:** 1 School of Art and Design, Xi’an University of Technology, Xi’an 710054, China; 2 Department of Applied Mathematics, Xi’an University of Technology, Xi’an 710054, China; 3 Design Art College, Xijing University, Xi’an 710123, China; 4 School of Computer Science and Engineering, Xi’an University of Technology, Xi’an 710048, China

**Keywords:** carnivorous plant algorithm, good point set, Cauchy mutation, differential evolution, engineering design

## Abstract

Many pivotal and knotty engineering problems in practical applications boil down to optimization problems, which are difficult to resolve using traditional mathematical optimization methods. Metaheuristics are efficient algorithms for solving complex optimization problems while keeping computational costs reasonable. The carnivorous plant algorithm (CPA) is a newly proposed metaheuristic algorithm, inspired by its foraging strategies of attraction, capture, digestion, and reproduction. However, the CPA is not without its shortcomings. In this paper, an enhanced multistrategy carnivorous plant algorithm called the UCDCPA is developed. In the proposed framework, a good point set, Cauchy mutation, and differential evolution are introduced to increase the algorithm’s calculation precision and convergence speed as well as heighten the diversity of the population and avoid becoming trapped in local optima. The superiority and practicability of the UCDCPA are illustrated by comparing its experimental results with several algorithms against the CEC2014 and CEC2017 benchmark functions, and five engineering designs. Additionally, the results of the experiment are analyzed again from a statistical point of view using the Friedman and Wilcoxon rank-sum tests. The findings show that these introduced strategies provide some improvements in the performance of the CPA, and the accuracy and stability of the optimization results provided by the proposed UCDCPA are competitive against all algorithms. To conclude, the proposed UCDCPA offers a good alternative to solving optimization issues.

## 1. Introduction

Recently, as technologies such as artificial intelligence, engineering design, urban transport planning, complex networks, and data processing have continued to develop, people are being faced with increasingly complex optimization problems [1,2]. It is very difficult and time-consuming to solve these problems with numerous variables and constraints because most optimization problems [3,4,5] in the real world have the following characteristics: large amounts of calculation, nonlinear constraints, nonconvexity, and a large and complex solution space [6]. Traditional optimization methods often struggle to solve these complex optimization problems and metaheuristic optimization algorithms have been introduced to overcome them [7]. These algorithms are capable of solving such complex problems in an iterative process. The low computational cost, flexibility, and simplicity of such algorithms have led to an increased interest among researchers in developing metaheuristic algorithms. Single-solution and population-based algorithms are the two broad groups into which metaheuristic algorithms fall. A single-solution algorithm creates a random solution and refines it until the best outcome is achieved; population-based algorithms produce a random collection of solutions and update those solutions throughout the course of iterations until the optimal answer is discovered. For a given problem, a single-solution-based algorithm may fall into a local optimum and thus fail to find the global optimum because it only generates and updates a random set of solutions. In contrast, population-based algorithms can find the global optimal solution by relying on information sharing because they generate a set of solutions, and they can eliminate local optimization. Therefore, population-based algorithms have become the focus of current research. Population-based algorithms can be divided into four categories [8]. They are based on evolution theory, natural phenomena, human activities, and swarm intelligence.

The evolutionary processes of reproduction, mutation, recombination, and selection serve as the basis for evolutionary algorithms. The genetic algorithm (GA) [9], one of the evolutionary theory-based algorithms, determines the best population by evolving the search space of a candidate population; the differential evolution algorithm (DE) [10] only requires a few simple control variables; and the symbiotic organism search (SOS) algorithm [11] imitates the symbiotic interaction tactics used by organisms in an ecosystem to survive and procreate.

Inspiration from natural phenomena can be divided into physics, chemistry, and biology. Physical laws such as gravity, electromagnetic force, inertia force, the heating and cooling of materials, etc., serve as inspiration for algorithms based on physical laws, such as the water cycle algorithm (WCA) [12], the multiverse optimizer (MVO) [13], and the black hole algorithm (BHO) [14], which simulates the attraction and absorption phenomena of black holes. Thermal diffusion served as an inspiration for simulated annealing (SA) [15]. The gravitational search algorithm (GSA) [16] is a recent algorithm that has been inspired by the Newtonian law of gravity and motion. Inspired by chemical reactions, the chemical reaction optimization algorithm (CRO) [17] recreates the chemical reaction of molecular interactions by reaching a low-energy stable state in the CRO. In terms of biology, based on the growth, division, and competition of bacteria in nature, Yang [18] suggested a network division approach. This method provides a uniform network divided into general processes. It is vital for the workload balance node.

Intelligent algorithms based on human activities include harmony search (HS) [19], teaching–learning-based optimization (TLBO) [20], and imperialist competition algorithm (ICA) [21]. All of these algorithms have their roots in human activities such as guitar tuning, educational practices, and imperial colonialism.

The swarm intelligence (SI) optimization method is a solution algorithm that was developed in accordance with the behavioral norms, survival criteria, and other mechanisms underlying the cooperative behavior of organisms or communities in nature. Because of its great effectiveness, straightforward structure, and straightforward execution, it has drawn the interest and research of many academics. Ant colony optimization (ACO) [22] is the SI optimization technique that best represents the field. There are many other algorithms such as the particle swarm optimization (PSO) [23], the gray wolf optimizer (GWO) [24], the sine cosine algorithm (SCA) [25], the whale optimization algorithm (WOA) [26], the moth–flame optimization algorithm (MFO) [27], hawks optimization (HHO) [28], the carnivorous plant algorithm (CPA) [29], and so on. Through information sharing and population cooperation, these algorithms encourage the evolution of the population toward the overall optimal goal. They simulate the foraging behavior of various populations and update individual behavior in a specific random manner. The application of intelligent algorithms is also extensive [30,31,32].

The CPA is a swarm intelligence optimization algorithm. The design of the CPA is inspired by carnivorous plants in nature. A model of the CPA was constructed by simulating the processes of predation, growth, and reproduction of carnivorous plants. When it comes to addressing frequent and complex optimization problems in a variety of sectors, the CPA algorithm has more clear advantages than other metaheuristic algorithms. However, the CPA method has several drawbacks, as do other optimization techniques. One of the CPA’s biggest drawbacks is that it tends to fall into local optimization and is not very good at exploring the search space.

This research proposes an enhanced CPA named UCDCPA, which enhances the performance and accuracy of the CPA. (1) The initial definition of the algorithm population by using the uniform initialization strategy of a good point set can effectively improve the uniformity of the distribution of the initial population, increase the effective area covered by the initial population in the whole feasible region, and increase the algorithm’s calculation precision and convergence speed. (2) The Cauchy mutation strategy is used for the initial population, which can increase the diversity of the initial population and reduce the probability of the leading offspring of the initial population falling into the local optimal solution. (3) Differential evolution is performed on the mixed population in the CPA algorithm. This enhances population variety, gives the algorithm mutation, crossover, and selection processes, and effectively lowers the likelihood that the algorithm would enter local optimization. The main contributions of this study are as follows:

- An enhanced CPA named UCDCPA is proposed. Three efficient strategies, i.e., good point set, Cauchy mutation, and differential evolution are applied to the UCDCPA to tackle the complex optimization tasks effectively.
- The performance of the UCDCPA is checked against the CEC2014 [33] and CEC2017 [34] test functions. The experimental results are compared with state-of-the-art algorithms, and some statistical analysis is carried out.
- The UCDCPA is applied to five classical engineering design problems. Specifically, pressure vessel design problems, welded beam design problems, tension/compression spring design (TCSD) problems, compound gear design problems, and cantilever structure problems. At the same time, some advanced algorithms are selected to compare their performance with the UCDCPA.

The remainder of the essay is structured as follows: The CPA algorithm is described in Section 2. The UCDCPA method is thoroughly explained in Section 3. In Section 4, real-world engineering optimization issues and numerical tests are used to demonstrate how well the UCDCPA performs in optimization. Finally, a brief summary of the information presented in this article is given and the next line of research is discussed.

## 2. Overview of the CPA

The CPA [29] simulates the process of predation by carnivorous plants, including the process through which carnivorous plants attract, capture, digest, and reproduce. The CPA starts by randomly initializing a set of solutions and divides the solutions into carnivorous plants and prey, then iterates according to the growth and reproduction process, and updates the fitness value in real time. The algorithm circulates the growth and reproduction process until the criteria for termination are satisfied. Each process is described in the following subsections.

### 2.1. Initialization

The CPA initializes a population with *n* individuals divided into carnivorous plants and prey as *nCPlant* and *nPrey*, respectively. Each individual is randomly initialized by Equation (1).

(1) $$Individual_{i,j}=Lb_{j}+(Ub_{j}-Lb_{j})\times rand$$

where $Ub_{j},Lb_{j}$ is the upper bound and lower bound of the *j*-th dimension of the individual, respectively, and *rand* is a random number in the range of [0,1].

A predetermined fitness function assesses each person’s fitness and the calculated fitness value is saved.

### 2.2. Classification and Grouping

The individuals are arranged in ascending order according to their fitness values (for the minimization problem). The first *nCPlant* individuals, after ranking, are carnivorous plant *CP*, and the remaining *nPrey* individuals were prey.

**Figure 1:**
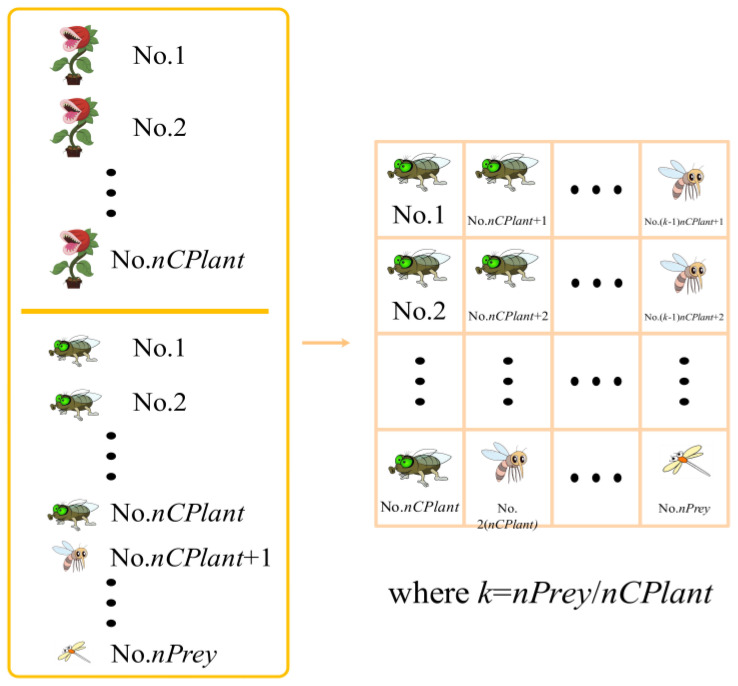
The grouping process of the CPA.

The top-ranked carnivorous plant receives the prey with the highest fitness value during the grouping phase. The second and third-ranked carnivorous plants, respectively, are given the second and third-ranked prey. The process is repeated until the carnivorous plant ranked *nCPlant* is assigned the prey ranked *nCPlant*. The visualization of the carnivorous plants and the prey is presented in Figure 1. On the left side of the picture, there are three different levels of prey, representing individuals of different qualities. The right side of the figure shows the grouping process of the algorithm.

### 2.3. Growth (Exploration)

Because of energy demand, carnivorous plants draw, capture, and consume prey. Prey are attracted by the plant’s aroma, but occasionally succeed in escaping the carnivorous plant’s control. As a result, an attraction rate is offered here.

Prey must be chosen at random by each group. The prey is caught and devoured by the carnivorous plant for growth if the attraction rate surpasses the random number of [0–1]. The model is as the following:

(2) $$NewCP_{i,j}=growth\times CP_{i,j}+(1-growth)\times {\mathrm{Prey}}_{v,j}$$

(3) growth=growth_rate×rand

where $CP_{i,j}$ refers to the carnivorous plant in rank *i*, and ${\mathrm{Prey}}_{v,j}$ is a randomly selected prey in group *i*. The attraction rate in the CPA is assigned as 0.8 for most cases.

Otherwise, if the attraction rate is smaller, the prey will be able to avoid the trap and survive.

(4) $${\mathrm{NewPrey}}_{i,j}=growth\times {\mathrm{Prey}}_{u,j}+(1-growth)\times {\mathrm{Prey}}_{v,j}\;u\neq v$$

(5) $$growth=\left\{\begin{array}{cc} growth\_rate\times rand & f({\mathrm{Prey}}_{v})>f({\mathrm{Prey}}_{u})\\ 1-growth\_rate\times rand & f({\mathrm{Prey}}_{v})<f({\mathrm{Prey}}_{u}) \end{array}\right.$$

where ${\mathrm{Prey}}_{u,j}$ is another randomly selected prey in the *i*th group. The growth process is repeated until it meets termination conditions.

### 2.4. Reproduction (Exploitation)

Only the best carnivorous plants in the CPA were permitted to procreate. This is to make sure that the CPA algorithm’s exploitation is focused on the best solution. The reproduction is described as follows:

(6) $$NewCP_{i,j}=CP_{1,j}+Reproduction\_rate\times rand\times mate_{i,j}$$

(7) $$mate_{i,j}=\left\{\begin{array}{cc} CP_{v,j}-CP_{i,j} & f(CP_{i})>f(CP_{v})\\ CP_{i,j}-CP_{v,j} & f(CP_{i})<f(CP_{v}) \end{array}\right.,i\neq v\neq 1$$

where $CP_{1,j}$ is the current best solution. During the reproduction process, the *v* is selected randomly for each dimension *j*.

### 2.5. Fitness Update and Combine the Population

A new group with a dimension is created when the newly formed population combines with the previous population. According to fitness values, the members of the new group are sorted in ascending order, and the top n individuals from the sorted group are chosen as new candidate solutions. This elitist selection approach makes sure that better solutions are chosen to be replicated in the following generation.

## 3. Improved Carnivorous Plant Algorithm

An enhanced carnivorous plant algorithm (UCDCPA) is proposed to enhance the CPA’s performance using three additional strategies: good point set uniform initialization, Cauchy mutation, and differential evolution. Firstly, the introduction of a good point set uniform initialization strategy improves the uniformity of initial population distribution and increases the effective area covered by the initial population in the whole feasible region. Secondly, a Cauchy mutation provides various opportunities for the initial population, increases the diversity of the initial population, and reduces the probability of the leading offspring of the initial population falling into the local optimal solution. Last but not least, the concept of differential evolution primarily addresses the issue that the CPA subgeneration population is prone to local optimization, offers mutation, crossover, and selection operations, and enhances the algorithm’s capacity to exit local optimization.

### 3.1. Good Point Set Uniform Initialization

The initial population in the CPA is produced at random, which theoretically has a uniform distribution; however, the distribution in the search space cannot be guaranteed to be uniform due to the impact of population size. The point set produced by the good point set method [35] has a greater distribution range and a low individual repetition rate, and it is equally dispersed over the search space. The good point set method can obtain a more stable, uniform, and wide initial population. The specific steps are as follows:

Step 1: Given that the size of the population is *n*, and *d* is the dimension of the problem.

(8) $$r_{j}=e^{j},1\leq j\leq d$$

Step 2: Based on Step 1, the points generated by the set of good points can be expressed as follows:

(9) $$r_{i,j}=i\cdot \left\lfloor i\cdot r_{j}\right\rfloor$$

Step 3: Introduce the good point set into population initialization, then the position of the individual is:

(10) $${Individual}_{i,j}=Lb_{j}+r_{i,j}(Ub_{j}-Lb_{j})$$

where 1 ≤ *j* ≤ *d*, *i* = 1, 2, …, *n*.

**Figure 2:**
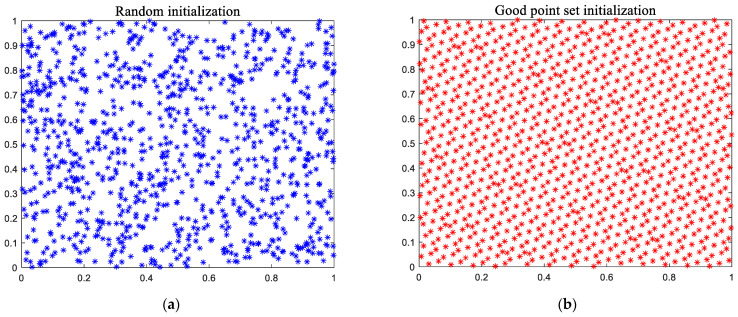
Two kinds of initialization affect expansion: (**a**) random initialization; and (**b**) good point set initialization.

Figure 2 depicts the distribution of coordinate points created in two-dimensional space using the random methods and the good point set method, and the number of coordinate points is 1000. Figure 2 shows that the coordinate points created by the good point set are more evenly distributed and there is no repetition, so the quality of the generated coordinate points is better.

### 3.2. Cauchy Mutation 

To extend the diversity of the population, the Cauchy mutation operation is carried out on the initial population so that the initial population can achieve a wider search range and improve the global optimization ability. The specific process of the Cauchy mutation [36] is as follows.

Cauchy mutation is based on the Cauchy probability density function, as described by Equation (11):

(11) $$g(x;x_{0},\gamma )=\frac{1}{\pi \gamma [1+{\left(\frac{x-x_{0}}{\gamma }\right)}^{2}]}$$

where $x_{0}$ is a positional parameter, γ is a random variable larger than 0, and *x* is a real number. In this article, *x*_0_ = 0 and γ = 1. This is the standard Cauchy distribution. By analyzing its probability density function, it can be seen that it has no specific mean and variance, but its mode and median are equal to the position parameter *x*_0_. The distribution function is shown in Equation (12):

(12) $$G(x)=\frac{1}{2}+\frac{1}{\pi }\arctan(x)$$

The Cauchy distribution is more uniform than the normal distribution, and the maximum value of the symmetry axis is gentler than the Gaussian distribution. Such distribution characteristics allow the Cauchy distribution to have great dispersion characteristics. The Cauchy mutation formula added in this paper is shown in Equations (13) and (14):

(13) $$X_{ibest}^{}=X_{i}^{}+X_{i}\times G(x)$$

(14) $$X_{i}=\left\{\begin{array}{cc} X_{ibest},f(X_{ibest}) & <f(X_{i})\\ X_{i},f(X_{ibest}) & \geq f(X_{i}) \end{array}\right.$$

### 3.3. Differential Evolution Theory

Differential evolution [10] is added to the CPA because the way that the CPA algorithm updates the population completely relies on the previous generation of individuals, and does not use the location information of the current generation of individuals. The specific steps of differential evolution are as follows:

#### 3.3.1. Operation of Mutation

Randomly select three individuals $X_{a}^{g},X_{b}^{g},X_{c}^{g}$, and the variation vector is generated by Equation (15)

(15) $$V_{i}^{g}=X_{a}^{g}+F\cdot (X_{b}^{g}-X_{c}^{g})$$

where F∈[0,2] is the scale factor.

#### 3.3.2. Cross Operation

The cross vector is generated by Equation (16)

(16) $$U_{i,j}^{g}=\left\{\begin{array}{l} V_{i,j}^{g},if(rand\leq CR,or,j=randi(n))\\ X_{i,j}^{g},otherwise \end{array}\right.,j=1,2,\cdots ,d$$

where *CR* is the cross rate, and *randi*(*n*) is a random integer of [1–N].

#### 3.3.3. Survival Criterion (Greedy Choice)

The vector with a better objective function value between the cross vector $U_{i}^{g}$ and $X_{i}^{g}$ will be used as the next-generation objective vector $X_{i}^{g+1}$.

(17) $$X_{i}^{g+1}=\left\{\begin{array}{l} U_{i}^{g},f(U_{i}^{g})\leq f(X_{i}^{g})\\ X_{i}^{g},f(U_{i}^{g})>f(X_{i}^{g}) \end{array}\right.$$

### 3.4. Detailed Description of the UCDCPA

By introducing good point set uniform initialization, Cauchy mutation, and differential evolution strategies into the CPA, the performance can be effectively improved. The following are the specific UCDCPA steps:

Step 1: Define the parameters of the UCDCPA.

Step 2: According to Equation (10), initialize the population.

Step 3: According to Equations (12)–(14), the Cauchy mutation is carried out on the initial population and the best one was selected for replacement.

Step 4: If the stop criteria are not met, loop through the following operations; otherwise, end the program.

Step 5: Classify and group the populations obtained after the Cauchy mutation in Step 3. Rank individuals according to fitness values. The former *nCPlant* individuals after the arrangement are regarded as carnivorous plants, and the remaining *nPrey* individuals are regarded as prey. The carnivorous plant ranked first in the grouping process receives the prey with the highest fitness value, and so on.

Step 6: If the attraction rate is greater than the generated random number, a new carnivorous plant individual is obtained according to Equations (2) and (3). On the contrary, new prey are generated according to Equations (4) and (5).

Step 7: The first carnivorous plant propagates its offspring according to Equations (6) and (7).

Step 8: Mix and sort the original population with the new population. Then, select the top *n* individuals as the new population.

Step 9: Three individuals, $X_{a}^{g},X_{b}^{g},X_{c}^{g}$, are randomly selected from the new species group, and the variation vector $V_{i}^{g}$ is obtained according to Equation (15).

Step 10: If *r* is less than the CR, or if *j* is a random integer equal to 1–*n*, cross vector $U_{i,j}^{g}=V_{i,j}^{g}$, otherwise $U_{i,j}^{g}=X_{i,j}^{g}$.

Step 11: Greedy selection of cross vector $U_{i}^{g}$ and current target vector $X_{i}^{g}$. Then go to Step 4.

**Figure 3:**
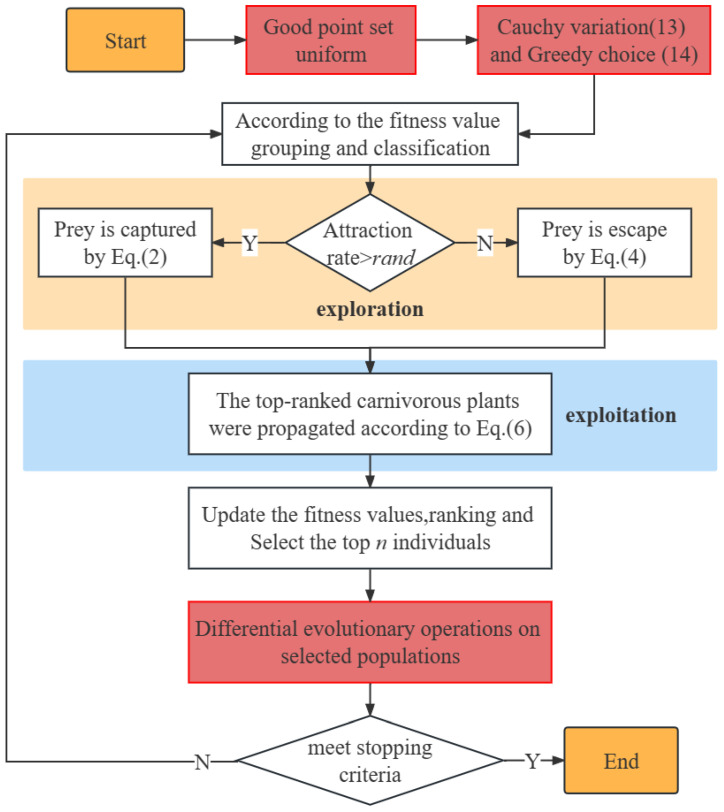
Flow chart of the UCDCPA.

The pseudo-code and the flow chart for the UCDCPA are given by Algorithm 1 and Figure 3, respectively.
**Algorithm 1**: The Pseudo code of the UCDCPADefine the parameters of the algorithm.Initialize a population of *n* individuals with *d* dimension randomly using Equations (8)–(10).A Cauchy mutation is performed on the initial population according to Equations (12)–(14).Sort the individuals by fitness value, and identify the best individual as g*.**Repeat until** the stopping condition is met. Classification and grouping of population individuals. **for** *i* = 1:*nCPlant*  **for** *Group_cycle* = 1:*group_iter*   **if** *attraction_rate* > *rand*    Equation (2)   **else**    Equation (4)  **end for** **end for** **for** *i* = 1:*nCPlant*  Equation (6) **end for** Evaluate the fitness of new individuals and combine the old and new populations Sort the individuals and select the top *n*-ranked individuals for the next generation. Differential evolution is carried out on the newly regenerated population by using Equations (15)–(17). Identify the current best individual g*.**end while.**

## 4. Numerical Experiment and Analysis

This section applies the UCDCPA algorithm to a set of test functions and demonstrates its superiority using several assessment indicators to prove the efficacy and stability of the UCDCPA algorithm in solving a variety of problems. The population is limited to 30, and there can be a maximum of 500 iterations. On test functions, each algorithm was executed 30 times. The operating environment is “Matlab 9.3.0.713579 on a 7th Gen Intel(R) Core(TM) i5-7200U CPU @ 2.50 GHz 2.71 GHz with 16 GB of RAM”.

Test functions are employed in this part to assess the UCDCPA’s performance. First, the CEC2014 test function [33] was tested. Second, to make the experimental results more persuasive, a test on the CEC2017 test function [34] is introduced to lessen the experiment’s randomness and contingency.

### 4.1. Parameter Setting

To assess the benefits and drawbacks of the UCDCPA, it is necessary to compare it with the CPA [29] and other representative algorithms, such as the arithmetic optimization algorithm [35], the coati optimization algorithm (COA) [36], the circle search algorithm (CSA) [37], COOT, the golden jackal optimization (GJO) algorithm [38], the rat swarm optimizer (RSO) [39], and the snake optimizer (SO) [40]. Some of the above algorithms’ parameter settings are presented in Table 1, while others have the same parameters as those found in the relevant literature.

### 4.2. Comparison of UCDCPA and Other Algorithms

To evaluate the effectiveness of the proposed UCDCPA and study the impact of good point set uniform initialization, Cauchy mutation, and differential evolution strategy on the CPA, UCDCPA, and CPA [29], the arithmetic optimization algorithm [35], the coati optimization algorithm (COA) [36], the circle search algorithm (CSA) [37], COOT, the golden jackal optimization (GJO) algorithm [38], the rat swarm optimizer (RSO) [39], and the snake optimizer (SO) [40] were compared.

Firstly, the performance of the UCDCPA and each algorithm was tested against the CEC2014 benchmark function. All algorithms make use of the same experimental parameters. The population is limited to 30, the problem’s dimension is 30, and there can be a maximum of 500 iterations. On test functions, each algorithm was executed 30 times. The test results for each algorithm based on the CEC2014 test function are shown in Table 2, which includes the best value, mean value, standard deviation (Std), and rank. The rank is given by the mean value and Std. First, the mean value is compared. When the mean value is the same, compare the Std. Items with smaller values have priority. The results are given according to the average rank of each algorithm. The end row of Table 2 displays the statistical outcomes of the Wilcoxon rank-sum test (significance level is 0.05) used to compare the other eight UCDCPA-based algorithms. “+” is the number of comparison algorithms that benefited the UCDCPA in terms of statistics, “=“ denotes the number of comparison algorithms that performed equally well, and “−” denotes other cases.

It can be seen from the data in Table 2 that our proposed UCDCPA algorithm can obtain the minimum value on 11 test functions, holds second place in the remaining 5 test functions, and there are 20 functions in the top three. The overall ranking is 3.30, which is in first place. On functions F3, F8, F12, and F16, the effect of the UCDCPA is not as good as that of the CPA, except for simple multimodal functions F12, the difference is minute, and the optimal value is very close. Among the eight composition functions of F23–F30, although the results are not as good as other comparison algorithms, they are better than those for the CPA. This shows that our improved algorithm has an obvious improvement effect. From the results of “+/=/−” in the table, compared with the CPA, the UCDCPA has great improvements in stability. Among the 30 functions, 20 are better than ARO, and 7 perform similarly; only 3 are worse than the CPA. In comparison with other algorithms, the UCDCPA also has obvious advantages.

**Figure 4:**
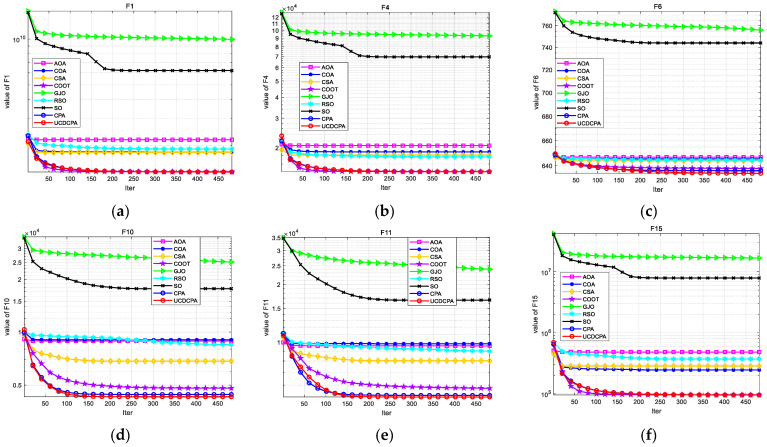

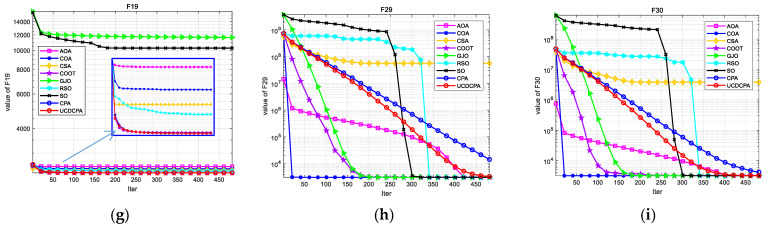
Convergence curves for all nine algorithms based on the results of the CEC2014 test function (**a**) F1; (**b**) F4; (**c**) F6; (**d**) F10; (**e**) F11; (**f**) F15; (**g**) F19; (**h**) F29; and (**i**) F30.

**Figure 5:**
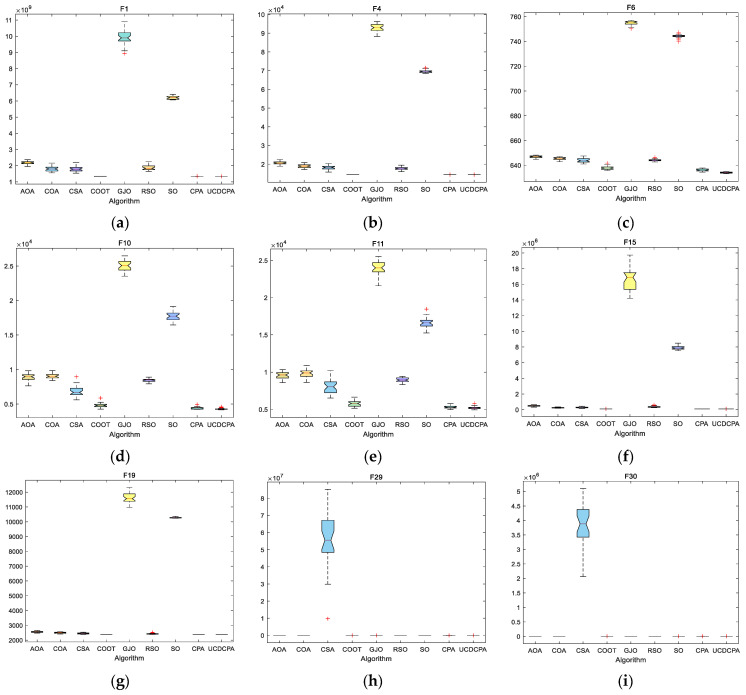
Boxplots for the nine algorithms based on the CEC2014 test function (**a**) F1; (**b**) F4; (**c**) F6; (**d**) F10; (**e**) F11; (**f**) F15; (**g**) F19; (**h**) F29; and (**i**) F30.

**Figure A1:**
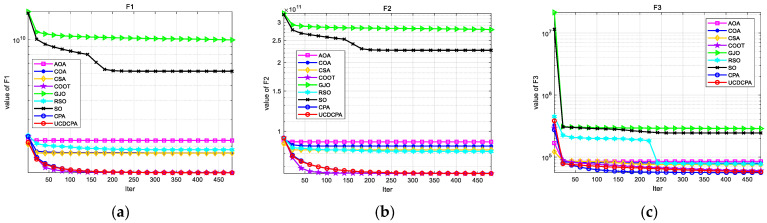

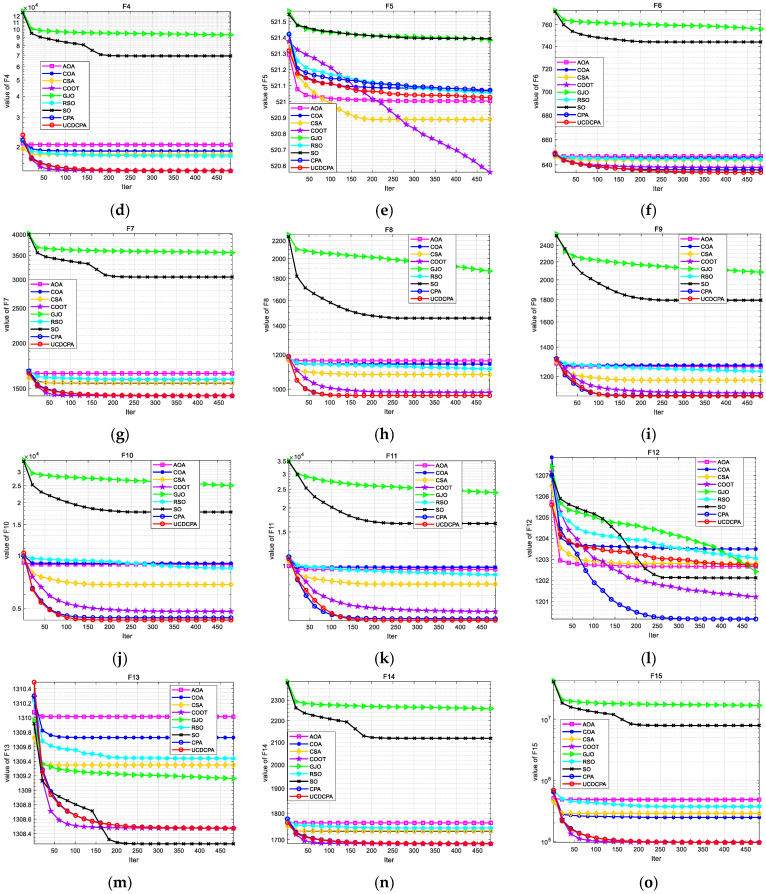

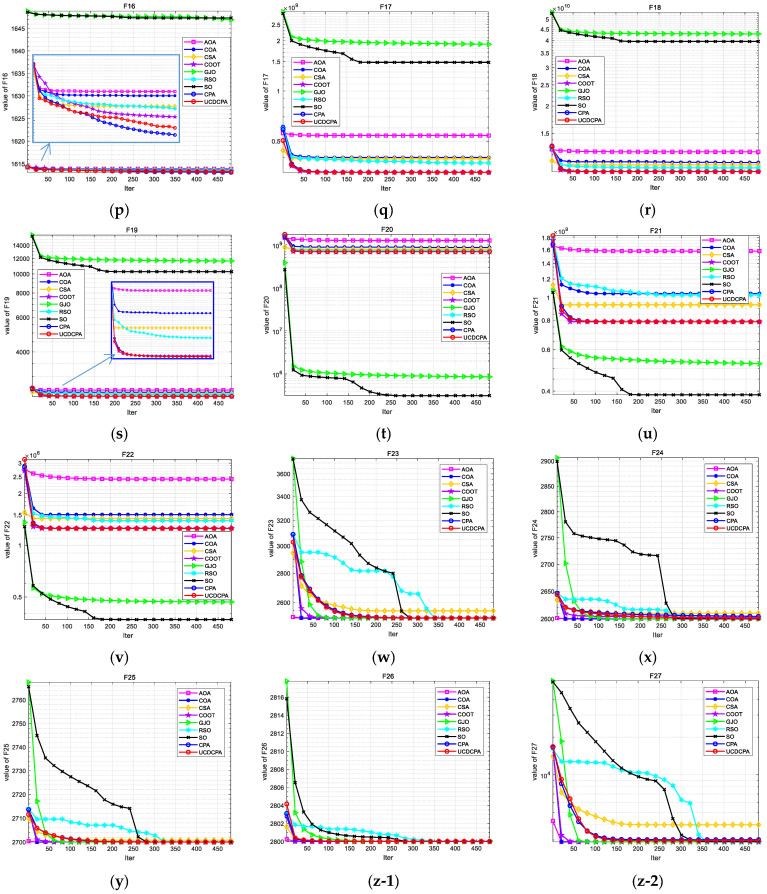

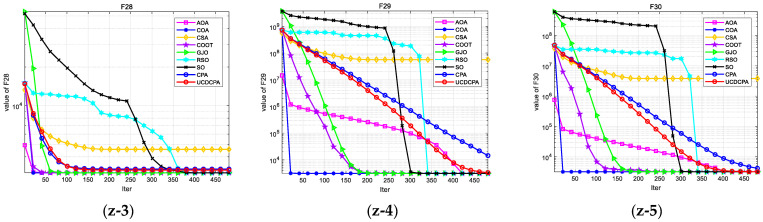
Convergence curves for nine algorithms based on the CEC2014 test function: (**a**) F1; (**b**) F2; (**c**) F3; (**d**) F4; (**e**) F5; (**f**) F6; (**g**) F7; (**h**) F8; (**i**) F9; (**j**) F10; (**k**) F11; (**l**) F12; (**m**) F13; (**n**) F14; (**o**) F15; (**p**) F16; (**q**) F17; (**r**) F18; (**s**) F19; (**t**) F20; (**u**) F21; (**v**) F22; (**w**) F23; (**x**) F24; (**y**) F25; (**z-1**) F26; (**z-2**) F27; (**z-3**) F28; (**z-4**) F29; and (**z-5**) F30.

**Figure A2:**
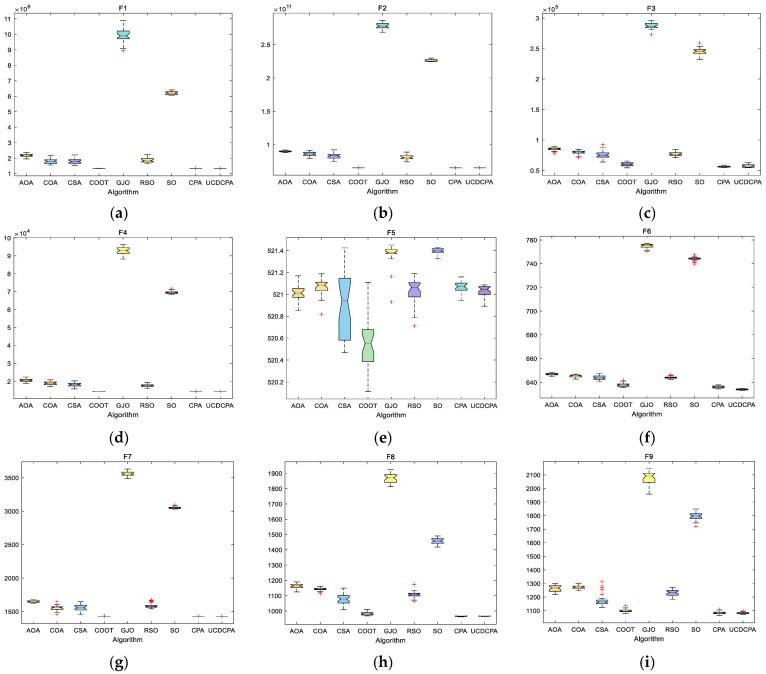

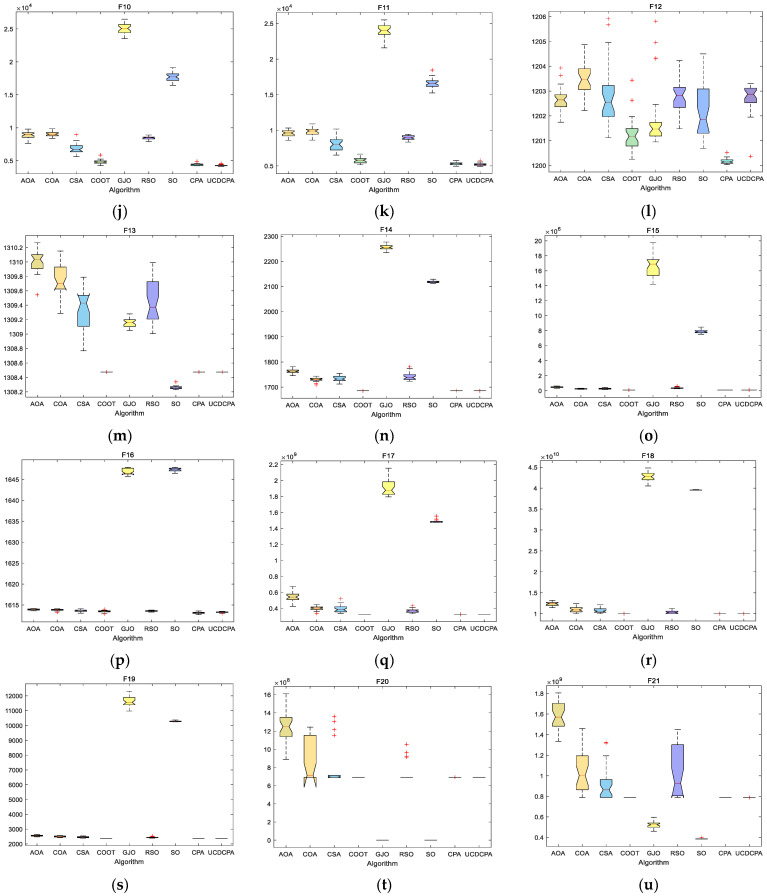

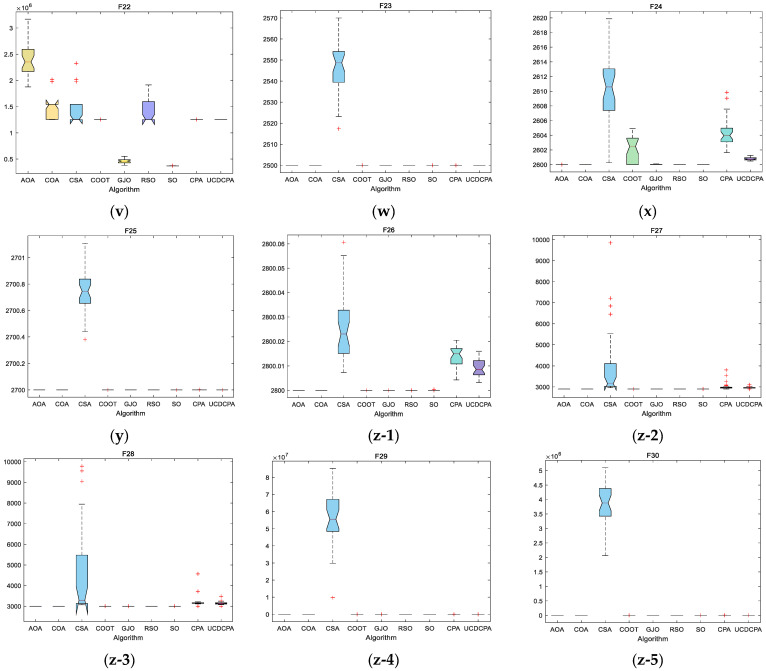
Boxplots for the nine algorithms based on the CEC2014 test function: (**a**) F1; (**b**) F2; (**c**) F3; (**d**) F4; (**e**) F5; (**f**) F6; (**g**) F7; (**h**) F8; (**i**) F9; (**j**) F10; (**k**) F11; (**l**) F12; (**m**) F13; (**n**) F14; (**o**) F15; (**p**) F16; (**q**) F17; (**r**) F18; (**s**) F19; (**t**) F20; (**u**) F21; (**v**) F22; (**w**) F23; (**x**) F24; (**y**) F25; (**z-1**) F26; (**z-2**) F27; (**z-3**) F28; (**z-4**) F29; and (**z-5**) F30.

To compare the nine algorithms more intuitively, to simplify the content and shorten the length of the article, Figure 4 and Figure 5 show the convergence curve (Average of 30 operation results) and boxplots of all algorithms on the same CEC2014 test sets. The complete convergence curve and boxplots can be found in Figure A1 and Figure A2. Figure 5 shows that the proposed UCDCPA has the best stability over the majority of the test functions. Combining the information in Table 2, it is clear that the proposed UCDCPA primarily enhances the stability and accuracy of the CPA. Since the original CPA can achieve values that are nearly optimal in some test functions, the proposed UCDCPA essentially accelerates the convergence rate of these test functions. In a comprehensive conclusion, the presented UCDCPA offers clear advantages over the other eight algorithms.

**Figure 6:**
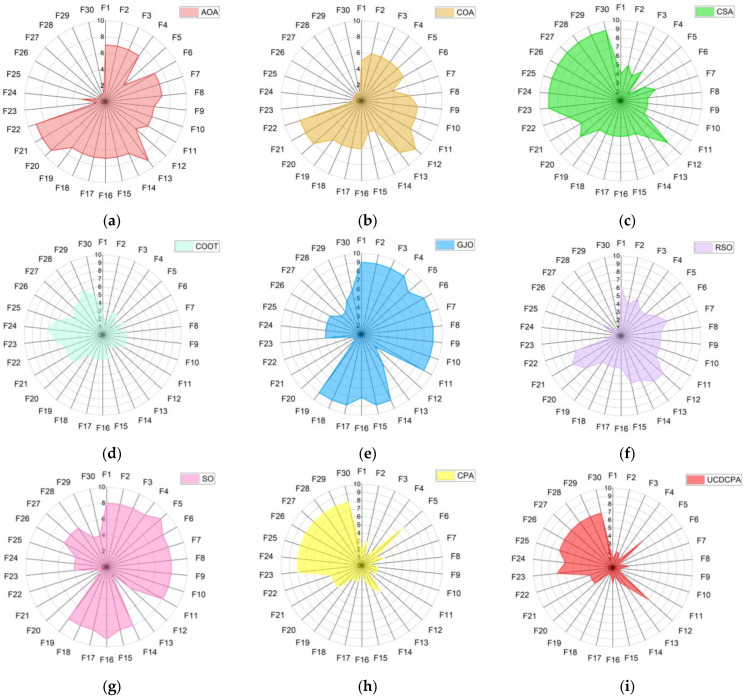
Radar diagrams for the nine algorithms based on the CEC2014 test function: (**a**) AOA; (**b**) COA; (**c**) CSA; (**d**) COOT; (**e**) GJO; (**f**) RSO; (**g**) SO; (**h**) CPA; and (**i**) UCDCPA.

To observe the ranking of each algorithm more intuitively, Figure 6 shows the ranking of all nine algorithms on 30 test functions. The smaller the area in the radar chart, the better the algorithm. It can be seen that the UCDCPA has the smallest area, so the UCDCPA performed the best against the CEC2014 test set.

The performance of each algorithm and UCDCPA was then evaluated once again using the CEC2017 benchmark function. The identical settings and experimental parameters as before were used. The test results for the nine algorithms on the CEC2017 test function are shown in Table 3.

From Table 3, we can see that our proposed UCDCPA can obtain the minimum value on 20 test functions, achieves second place in the remaining 6 test functions, and is in the top three on 28 test functions, with an overall ranking of 1.45, ranking first. In the seventh, simple multimodal test functions F4–F10, only on F9 the UCDCPA was less effective than the CPA and won first place in five test functions. In the hybrid test functions F11–F20, the UCDCPA was superior to the CPA, with an obvious improvement effect. Eight of the ten functions reach first place. In the set of composition functions, the UCDCPA embodies the advantage, with 70% of functions being first, and 100% of functions being in the top two, which fully demonstrates the UCDCPA’s ability in the computational processing of composition functions. Overall, our proposed UCDCPA has advantages over the nine algorithm species and outperforms the comparison algorithms. From the results of “+/=/−” in the table, compared with the CPA, the UCDCPA has great improvements in stability. Among the 29 functions, 15 are better than the CPA, and 13 perform similarly; only one is worse than the CPA. In comparison with other algorithms, the UCDCPA also has obvious advantages.

**Figure 7:**
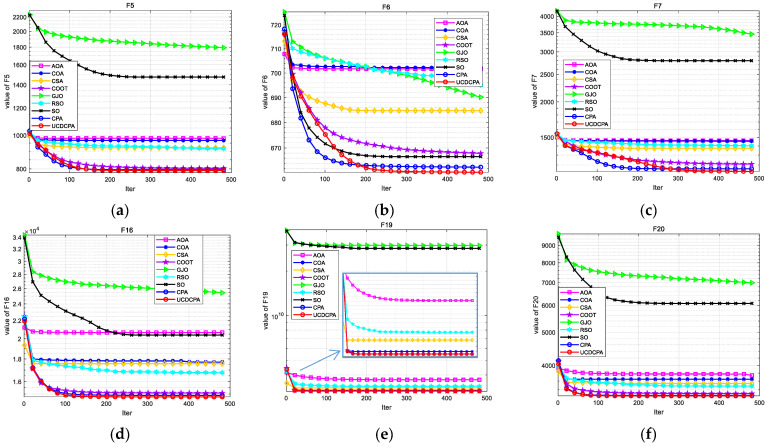

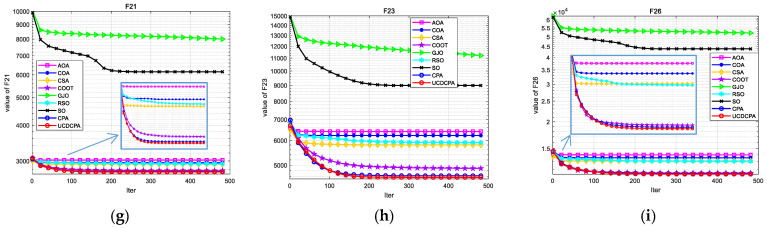
Convergence curves for all nine algorithms based on the CEC2017 test function: (**a**) F5; (**b**) F7; (**c**) F7; (**d**) F16; (**e**) F19; (**f**) F20; (**g**) F21; (**h**) F23; and (**i**) F26.

**Figure 8:**
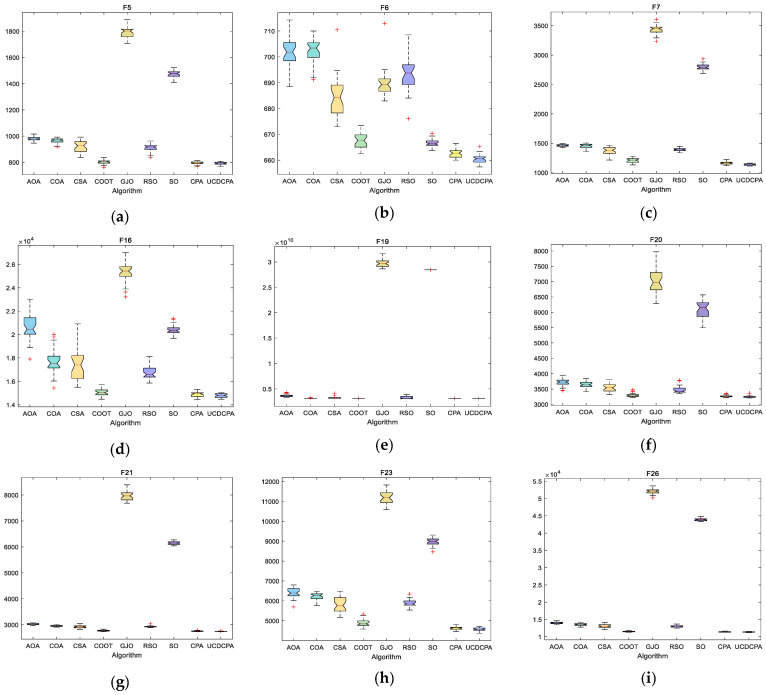
Boxplots for the nine algorithms based on the CEC2017 test function: (**a**) F5; (**b**) F7; (**c**) F7; (**d**) F16; (**e**) F19; (**f**) F20; (**g**) F21; (**h**) F23; and (**i**) F26.

**Figure A3:**
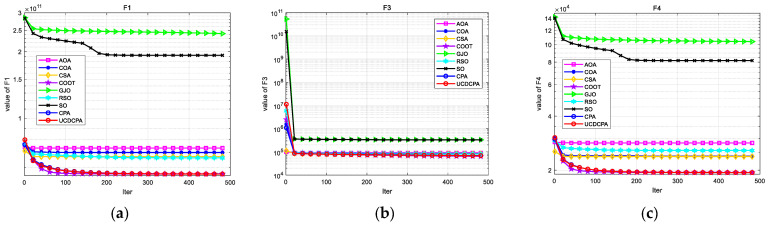

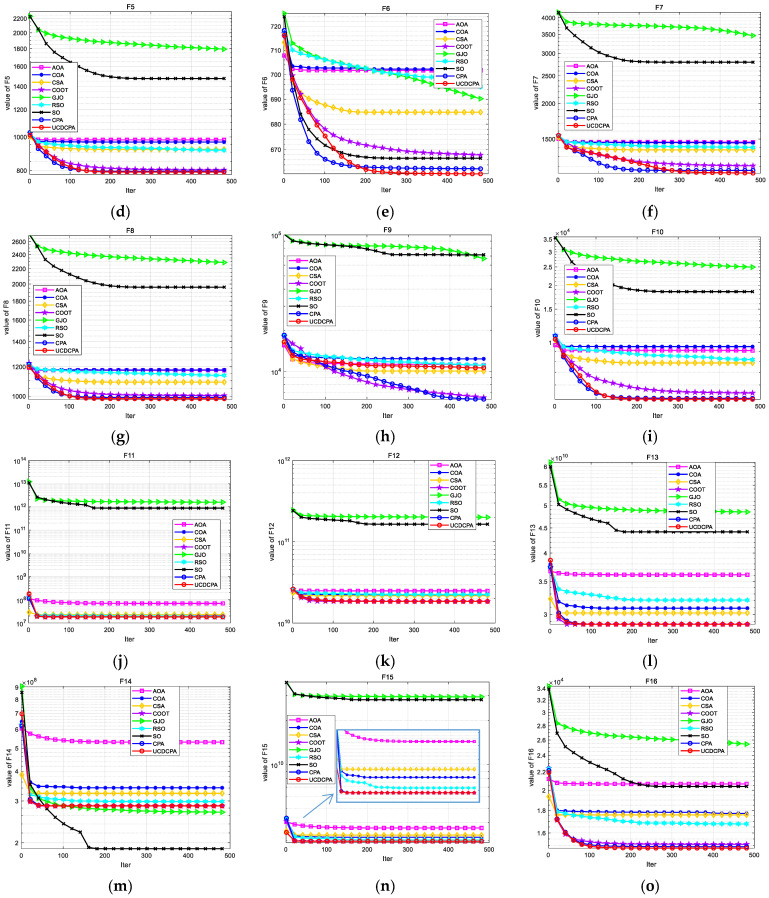

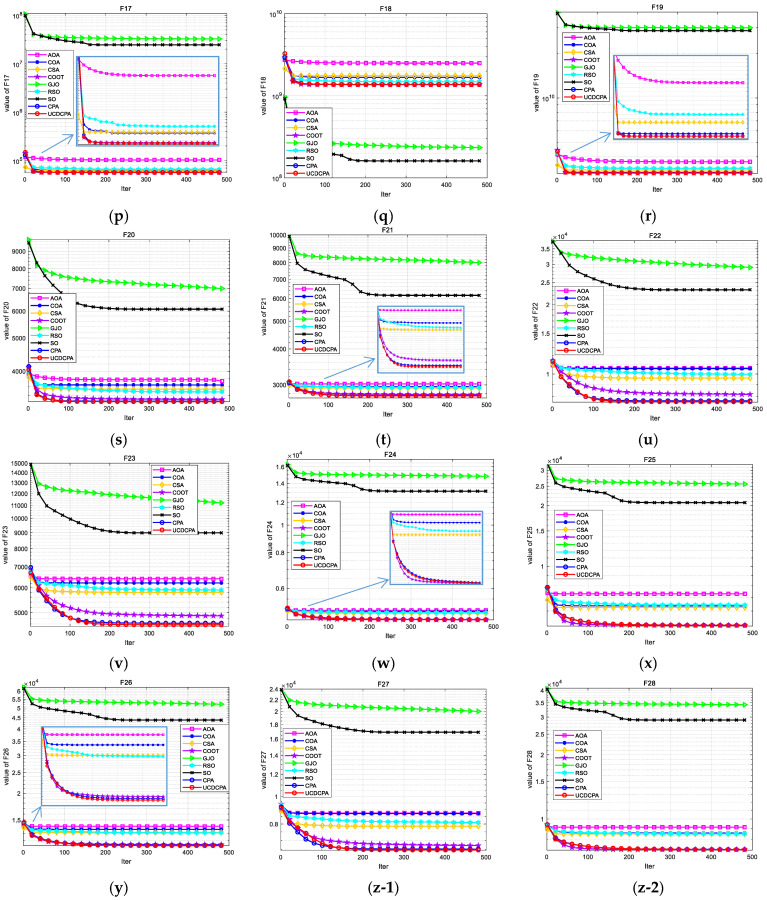

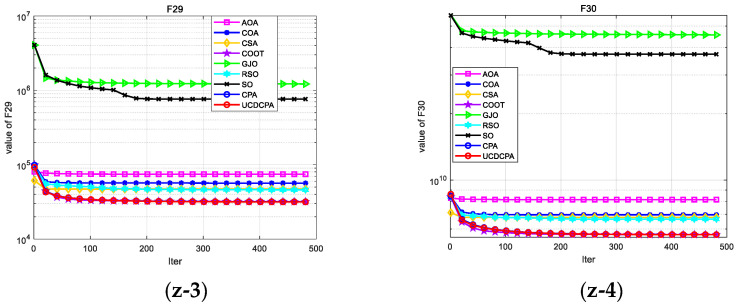
Convergence curves for nine algorithms based on the CEC2017 test function: (**a**) F1; (**b**) F3; (**c**) F4; (**d**) F5; (**e**) F6; (**f**) F7; (**g**) F8; (**h**) F9; (**i**) F10; (**j**) F11; (**k**) F12; (**l**) F13; (**m**) F14; (**n**) F15; (**o**) F16; (**p**) F17; (**q**) F18; (**r**) F19; (**s**) F20; (**t**) F21; (**u**) F22; (**v**) F23; (**w**) F24; (**x**) F25; (**y**) F26; (**z-1**) F27; (**z-2**) F28; (**z-3**) F29; and (**z-4**) F30.

**Figure A4:**
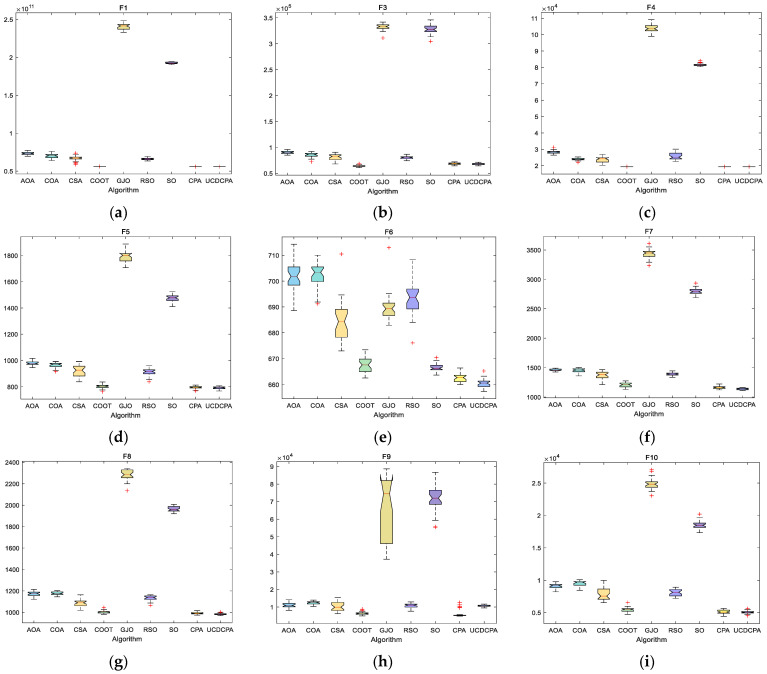

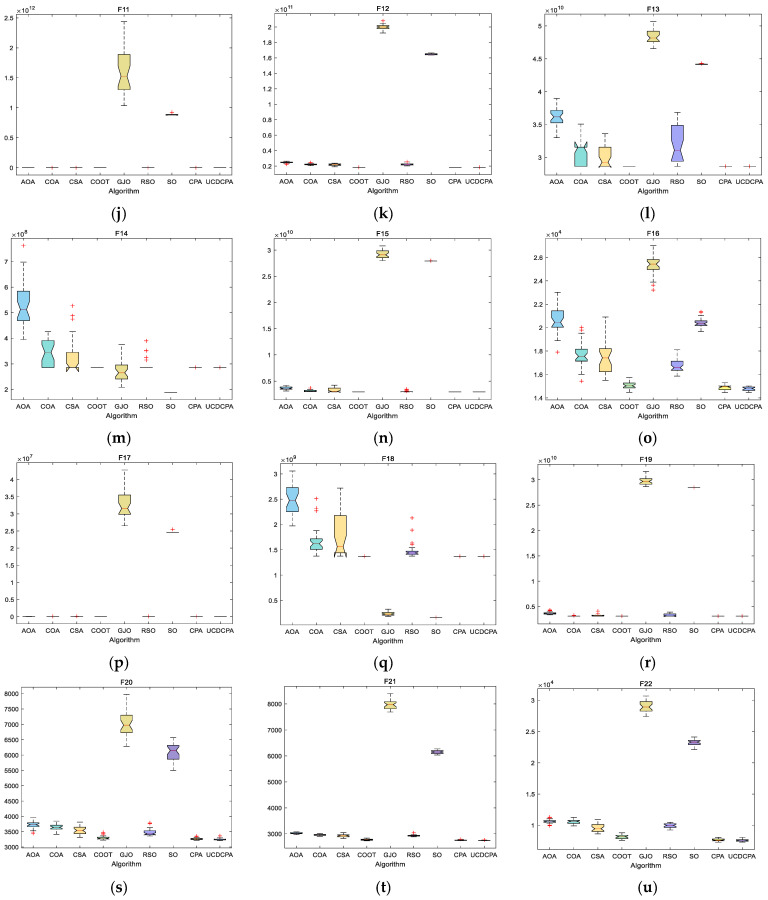

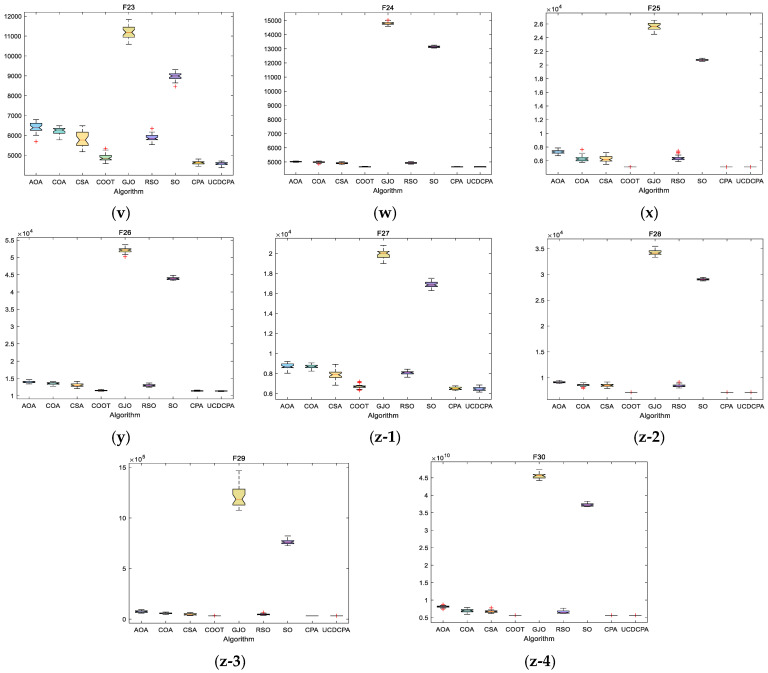
Boxplots for the nine algorithms based on the CEC2017 test function: (**a**) F1; (**b**) F3; (**c**) F4; (**d**) F5; (**e**) F6; (**f**) F7; (**g**) F8; (**h**) F9; (**i**) F10; (**j**) F11; (**k**) F12; (**l**) F13; (**m**) F14; (**n**) F15; (**o**) F16; (**p**) F17; (**q**) F18; (**r**) F19; (**s**) F20; (**t**) F21; (**u**) F22; (**v**) F23; (**w**) F24; (**x**) F25; (**y**) F26; (**z-1**) F27; (**z-2**) F28; (**z-3**) F29; and (**z-4**) F30.

To visually compare the nine algorithms, the convergence curves (average of 30 operation results) of the nine algorithms in the same CEC2017 test functions are given in Figure 7, and the boxplots of each algorithm in the same CEC2017 test functions are given in Figure 8. The complete convergence curve and boxplots can be found in Figure A3 and Figure A4. From Figure 8, we can see that the UCDCPA has a more obvious advantage in stability compared to the CPA and others. Combining Table 3 and Figure 7, it is clear that the proposed UCDCPA primarily enhances computational correctness when compared to the CPA, due to the CPA already achieving high convergence speed on some test functions, such as F6, F16, and F19. A thorough analysis reveals that the suggested UCDCPA algorithm has several benefits over the other eight methods.

**Figure 9:**
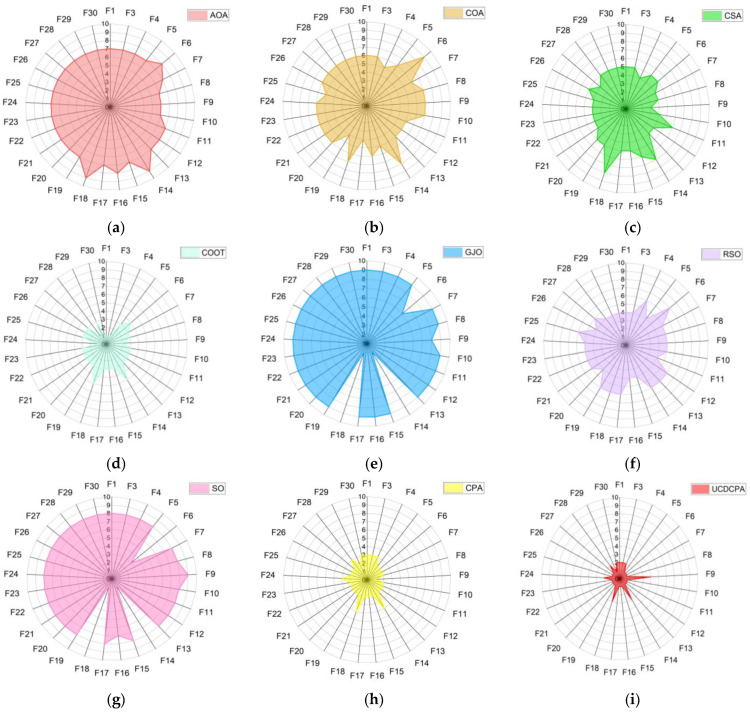
Radar diagram for nine algorithms based on the CEC2017 test function: (**a**) AOA; (**b**) COA; (**c**) CSA; (**d**) COOT; (**e**) GJO; (**f**) RSO; (**g**) SO; (**h**) CPA; and (**i**) UCDCPA.

Figure 9 shows the ranking of the nine algorithms on 29 test functions of CEC2017. Consistent with the results on the CEC2014 test set, on the CEC2017 test set, the UCDCPA again has the smallest area and the best performance, gaining advantages over all nine algorithms.

## 5. Improved Carnivorous Plant Algorithm (UCDCPA) for Engineering Design

### 5.1. Pressure Vessel Design Problems

**Figure 10:**
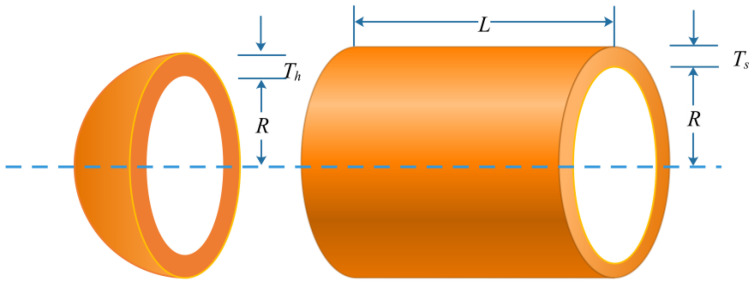
Diagram illustrating the pressure vessel issue.

The primary objective of the pressure vessel design [41] is to reduce the cost of the vessel’s materials, forming, and welding. As shown in Figure 10, the four variables in this problem are the inner radius (*R*), the length of the cylindrical section without taking the head into account (*L*), the thickness of the head (*Th*), and the thickness of the shell (*Ts*). The four constraint functions and the problem’s mathematical formulation are provided in Equation (18).

(18) $$\begin{array}{cc} \min f= & 19.84T_{s}^{2}R+1.7781T_{h}R^{2}+0.6224T_{s}Rx_{4}+3.1661T_{s}^{2}L\\ \mathrm{Meet}\;\mathrm{to} & 0.0193R\leq T_{s}\\ & -T_{h}+0.00954R\leq 0\\ & \pi R^{2}L+\frac{4}{3}\pi R^{3}+\geq 1.296.000\\ & L\leq 240\\ \mathrm{where} & 0\leq T_{s},T_{h}\leq 100;10\leq R,L\leq 200. \end{array}$$

The proposed UCDCPA was used to deal with the pressure vessel design problem, and the results were compared to the CPA and the other 12 algorithms. In Table 4, each algorithm’s minimum cost and related variable values are displayed, and it can be seen that the UCDCPA works better. Table 5 illustrates the statistical findings for each algorithm, with the UCDCPA outperforming the others in terms of the best solution, average solution, worst solution, and standard deviation. A smaller standard deviation suggests that an algorithm is more robust. This leads to the conclusion that, in comparison to competing algorithms, the proposed UCDCPA offers a competitive advantage.

### 5.2. Welded Beam Design Problem

**Figure 11:**
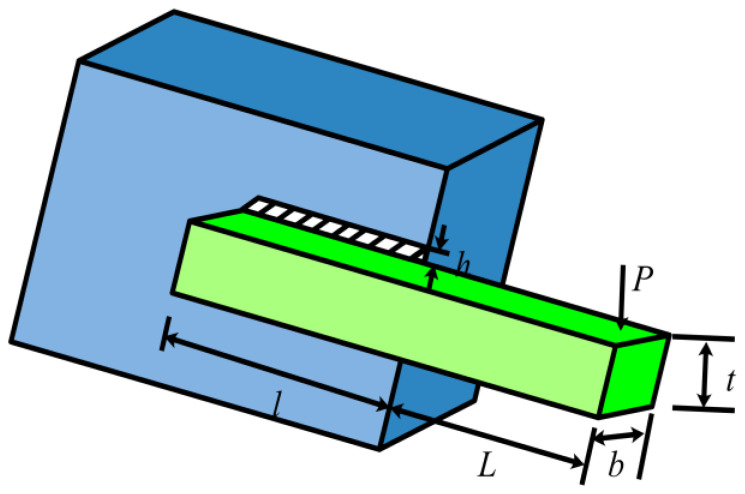
Diagram of the welded beam issue.

The welded beam design problem aims to determine the lowest fabrication cost for a welded beam [46]. The length of the clamped bar (*l*), the height of the bar (*t*), the thickness of the bar (*b*), and the thickness of the weld (*h*) are the four design factors that need to be optimized, as shown in Figure 11. In addition, seven constraints are to be satisfied by applying loads to the top of the bars. The specific design problem is shown in Equation (19).

(19) $$\begin{array}{cc} \min\;f= & 0.04811tb(l+14)+1.10471h^{2}l\\ \mathrm{subject}\;\mathrm{to}\; & \tau ([h,l,t,b])\leq \tau_{\max}\\ & \sigma ([h,l,t,b])\leq \sigma_{\max}\\ & \delta ([h,l,t,b])\leq \delta_{\max}\\ & h\leq b\\ & P\leq P_{c}([h,l,t,b])\\ & 0.125\leq h\\ & 0.04811tb(l+14)\leq 0.5-1.10471h^{2}\\ \mathrm{where}\;0.1 & \leq h,b\leq 2;0.1\leq l,t\leq 10; \end{array}$$

The welded beam design problem has been solved using the presented UCDCPA, and its performance was evaluated against that of the CPA and the other 12 intelligent algorithms. For each method, the minimum costs and accompanying ideal variable values are listed in Table 6. The GWO, PSO, and CPA algorithms all calculate good results, and the minimum cost of the CPA is consistent with the optimal cost of the UCDCPA, but the proposed UCDCPA is better. Table 7 displays the statistical outcomes for all methods. The standard deviation of the UCDCPA is zero, indicating that the algorithm has strong robustness. The best solution, average solution, worst solution, and standard deviation obtained using the UCDCPA are all better than those obtained using other algorithms. This leads to the conclusion that the proposed UCDCPA has a competitive advantage over existing algorithms in tackling this problem in every scenario.

### 5.3. Tension/Compression Spring Design (TCSD) Problem

**Figure 12:**
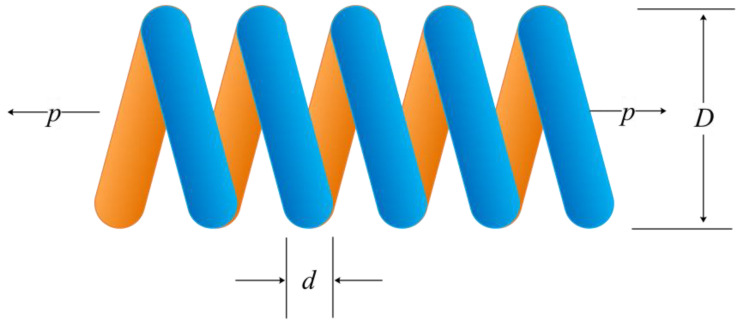
Diagram showing the tension/compression spring.

Finding the values of the three parameters of the wire diameter (*u*_1_), mean coil diameter (*u*_2_), and the number of effective coils (*u*_3_) is the goal of the TCSD problem [47] (see Figure 12). The TCSD problem is described mathematically in Equation (20).

(20) $$\begin{array}{cc} \mathrm{Min}\;f(\vec{u}) & =(u_{3}+2)u_{2}u_{1}^{2}\\ \mathrm{sub}\;g_{1}(\vec{u}) & =1-\frac{u_{2}^{3}u_{3}}{71785u_{1}^{4}}\leq 0\\ g_{2}(\vec{u}) & =\frac{4u_{2}^{2}-u_{1}u_{2}}{12566(u_{2}u_{1}^{3}-u_{1}^{4})}+\frac{1}{5108u_{1}^{2}}\leq 0\\ g_{3}(\vec{u}) & =1-\frac{140.45u_{1}}{u_{2}^{2}u_{3}}\leq 0\\ g_{4}(\vec{u}) & =\frac{u_{1}+u_{2}}{1.5}-1\leq 0\\ Variables\;range & 0.05\leq u_{1}\leq 2\\ & 0.25\leq u_{2}\leq 1.30\\ & 2.00\leq u_{3}\leq 15 \end{array}$$

The minimum cost and accompanying ideal variable values for each algorithm used to solve the TCSD issue are shown in Table 8. The ALO, CPA, and the UCDCPA all show good results. Table 9 displays the statistical outcomes for all methods. The UCDCPA’s calculations of the best solution, average solution, worst solution, and standard deviation outperform those of the other methods, and the standard deviation, with a significant advantage, illustrates that the UCDCPA has better robustness. According to the thorough investigation, the proposed UCDCPA has a considerable competitive edge over other algorithms in resolving this problem.

### 5.4. Compound Gear Design Problem

**Figure 13:**
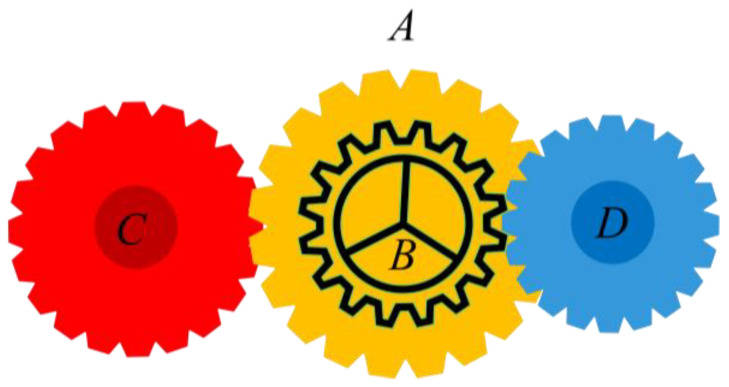
Compound gear design problem.

A mechanical engineering problem, the “gear train design problem” [50], seeks to reduce the ratio of a particular gear set, denoted as (*n_B_n_D_*)/(*n_C_n_A_*). As can be seen from Figure 13, the four parameters of this problem are the number of teeth of the gears, which are integers and range in size from 12 to 60. As a result, the issue of gear train design is discrete. The variables’ ranges are regarded as constraints.

(21) $$\begin{array}{l} \min f(x)={\left(\frac{1}{6.931}-\frac{x_{3}x_{2}}{x_{1}x_{4}}\right)}^{2}\\ \mathrm{subject}\;\mathrm{to}\text{:}\\ 12\leq x_{i}\leq 60\;i=1,2,\cdots ,4 \end{array}$$

To solve the gear train design challenge, the original algorithm and the other 13 clever algorithms are compared to the proposed UCDCPA. The minimum cost and accompanying ideal variable values for each method are displayed in Table 10, and the results obtained using the GSA, AOA, AO, SCA, GWO, PSO, WHO, MVO, CPA, and the UCDCPA algorithms are similar. Table 11 displays all algorithms’ statistical results. The best, mean, worst, and Std. obtained using the UCDCPA algorithm are better than others, and the algorithm’s standard deviation, with considerable advantage, shows that it is more robust than other methods.

### 5.5. Cantilever Structure Problem

**Figure 14:**
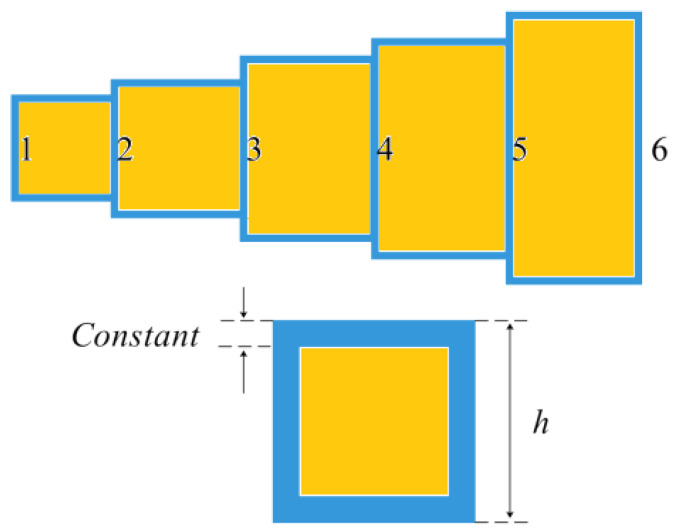
Structural drawing of the cantilever beam.

The cantilever structure problem is a structural engineering design problem [44]. In this application, the goal is to reduce a cantilever’s weight while taking into account load-bearing capacity limits (see Figure 14). The choice variables are the heights of the five hollow squares that make up the beam.

(22) $$\begin{array}{l} \mathrm{Min}\;f=0.06224(h_{1}+h_{2}+h_{3}+h_{4}+h_{5}),\\ \mathrm{subject}\;\mathrm{to}\;g=\frac{61}{h_{1}^{3}}+\frac{27}{h_{2}^{3}}+\frac{19}{h_{3}^{3}}+\frac{7}{h_{4}^{3}}+\frac{1}{h_{5}^{3}}-1\leq 0,\\ \mathrm{meet}\;0.01\leq h_{i}\leq 100\;i=1,2,\cdots ,5 \end{array}$$

Table 12 shows that there is not much difference between the results calculated using the UCDCPA and the CPA, with the UCDCPA slightly outperforming the CPA. Table 13 compares the statistical outcomes of this algorithm with other approaches. As shown in the table, although the solutions obtained using the CPA are highly competitive, the UCDCPA is slightly better than the CPA when the best, mean, worst, and Std. of the results are considered together. The effectiveness of the improved algorithm proposed in this paper is illustrated.

## 6. Conclusions

In this paper, an improved carnivorous plant algorithm (UCDCPA) is proposed based on the CPA. After carefully analyzing the process of the CPA, three strategies were introduced to enhance the performance of the CPA. First, the initialization of the population was completed using the good point set. Secondly, the Cauchy mutation method was used to increase the initial population diversity. Finally, a differential evolution strategy with good exploration ability was integrated into the CPA. Numerical tests were conducted on a total of 59 benchmark functions to test the performance of the proposed UCDCPA. The test results show that the UCDCPA outperforms the CPA and seven other novel metaheuristics. To further validate the optimization capability of the UCDCPA, five real engineering problems were tested. The test results further validate the high performance of the UCDCPA in solving real-world problems. The future work direction is to extend the UCDCPA to multiobjective optimization, which can also be applied to training neural networks, computer graphics, or other problem fields [52,53].

## Figures and Tables

**Table 1:** Algorithm parameter setting.

Algorithm	Parameter	Set Value
SSA	Proportion of producers	*P_percent* = 0.2
CPA	Each rate setting	*attraction_rate* = 0.8
		*growth_rate* = 2
		*reproduction_rate* = 1.8
UCDCPA	Variation rate	$F=0.5\times 2\times \exp(1-\frac{Gm}{Gm+1-G})$
	Cross rate	*CR* = 0.9

**Table 2:** The results of each algorithm based on the CEC2014 test function set.

F	Index	Algorithm								
		AOA	COA	CSA	COOT	GJO	RSO	SO	CPA	UCDCPA
F1	Best	1.9514E+09	1.5536E+09	1.5395E+09	1.3384E+09	8.9338E+09	1.6496E+09	6.0687E+09	1.3384E+09	1.3384E+09
	Mean	2.1804E+09	1.8037E+09	1.7997E+09	1.3416E+09	9.9229E+09	1.8955E+09	6.2075E+09	1.3387E+09	1.3385E+09
	Std	9.0926E+07	1.6225E+08	1.7284E+08	2.9883E+06	4.8937E+08	1.8298E+08	1.0778E+08	5.5732E+05	7.4499E+04
	Rank	7	5	4	3	9	6	8	2	1
F2	Best	8.8122E+10	7.9635E+10	7.4658E+10	6.5317E+10	2.6807E+11	7.4235E+10	2.2424E+11	6.5325E+10	6.5324E+10
	Mean	8.9885E+10	8.6069E+10	8.2928E+10	6.5317E+10	2.7781E+11	8.1317E+10	2.2602E+11	6.5340E+10	6.5335E+10
	Std	1.0436E+09	3.5636E+09	4.3173E+09	4.0629E+02	4.6046E+09	3.5123E+09	1.4763E+09	1.2301E+07	6.7195E+06
	Rank	7	6	5	1	9	4	8	3	2
F3	Best	7.8285E+04	7.1451E+04	6.3714E+04	5.4267E+04	2.7270E+05	7.0994E+04	2.3188E+05	5.4378E+04	5.4426E+04
	Mean	8.5159E+04	7.9614E+04	7.5663E+04	6.0061E+04	2.8741E+05	7.6962E+04	2.4460E+05	5.6205E+04	5.7388E+04
	Std	2.7135E+03	3.4274E+03	6.8448E+03	3.2032E+03	5.5788E+03	3.3212E+03	6.5889E+03	1.0904E+03	2.4630E+03
	Rank	7	6	4	3	9	5	8	1	2
F4	Best	1.8941E+04	1.7133E+04	1.5770E+04	1.4415E+04	8.8229E+04	1.5961E+04	6.8574E+04	1.4415E+04	1.4415E+04
	Mean	2.0624E+04	1.8901E+04	1.8105E+04	1.4421E+04	9.2630E+04	1.7664E+04	6.9590E+04	1.4417E+04	1.4416E+04
	Std	7.5524E+02	9.9267E+02	1.1830E+03	6.8554E+00	2.3244E+03	8.7779E+02	6.8663E+02	1.8640E+00	1.0466E+00
	Rank	7	6	5	3	9	4	8	2	1
F5	Best	520.85	5.21E+02	5.20E+02	520.11	520.93	520.71	521.33	520.94	520.89
	Mean	521.00	5.21E+02	5.21E+02	520.54	521.37	521.02	521.39	521.07	521.03
	Std	0.08	8.03E−02	3.07E−01	0.23	0.10	0.11	0.03	0.05	0.06
	Rank	3	6	2	1	8	4	9	7	5
F6	Best	644.73	642.78	641.01	635.73	750.17	642.61	739.72	634.42	633.06
	Mean	646.80	645.42	643.86	637.83	754.97	644.17	744.09	636.09	634.03
	Std	0.96	1.14	1.76	1.49	1.74	0.92	1.56	0.94	0.50
	Rank	7	6	4	3	9	5	8	2	1
F7	Best	1617.92	1460.07	1451.98	1430.41	3488.85	1540.79	3032.47	1430.46	1430.45
	Mean	1650.31	1551.33	1557.37	1430.53	3559.75	1588.44	3050.75	1430.56	1430.49
	Std	16.43	38.04	47.02	0.34	34.71	39.85	14.61	0.11	0.03
	Rank	7	4	5	2	9	6	8	3	1
F8	Best	1124.28	1112.08	1009.86	967.87	1812.76	1063.44	1418.59	961.85	963.65
	Mean	1161.42	1142.87	1080.79	982.72	1867.41	1108.18	1457.42	966.28	966.36
	Std	18.94	10.73	38.60	11.22	30.18	19.77	19.49	1.53	1.29
	Rank	7	6	4	3	9	5	8	1	2
F9	Best	1219.58	1246.68	1124.28	1080.42	1958.59	1183.55	1720.72	1064.84	1073.25
	Mean	1264.48	1273.83	1176.37	1098.62	2080.52	1232.56	1795.39	1084.63	1082.07
	Std	21.45	13.73	46.15	12.42	48.01	21.14	26.53	10.23	6.08
	Rank	6	7	4	3	9	5	8	2	1
F10	Best	7644.20	8397.83	5607.98	4252.20	23,535.81	7926.79	16,452.52	4218.94	4144.42
	Mean	8880.93	9035.39	6833.78	4804.01	25,043.01	8469.02	17,701.11	4420.90	4289.61
	Std	515.89	345.36	761.04	309.82	807.52	248.72	684.07	182.88	114.29
	Rank	6	7	4	3	9	5	8	2	1
F11	Best	8593.04	8622.52	6516.24	5151.18	21,583.81	8337.22	15,249.30	4955.96	4936.69
	Mean	9575.61	9830.41	8029.47	5772.96	23,926.04	8959.48	16,580.37	5308.01	5206.60
	Std	489.65	515.46	902.15	407.17	1062.96	307.49	677.47	197.94	176.11
	Rank	6	7	4	3	9	5	8	2	1
F12	Best	1201.74	1202.22	1201.12	1200.26	1200.95	1201.49	1200.69	1200.03	1200.37
	Mean	1202.65	1203.49	1202.80	1201.19	1201.87	1202.74	1202.12	1200.17	1202.72
	Std	0.48	0.61	1.29	0.67	1.25	0.66	0.99	0.11	0.58
	Rank	5	9	8	2	3	7	4	1	6
F13	Best	1309.54	1309.29	1308.77	1308.47	1309.05	1309.00	1308.23	1308.47	1308.47
	Mean	1310.01	1309.73	1309.35	1308.47	1309.15	1309.43	1308.26	1308.48	1308.47
	Std	0.15	0.23	0.27	0.00	0.06	0.28	0.02	0.00	0.00
	Rank	9	8	6	2	5	7	1	4	3
F14	Best	1745.65	1708.54	1712.35	1685.40	2234.88	1723.59	2112.58	1685.43	1685.42
	Mean	1763.24	1730.73	1733.38	1685.40	2256.98	1743.86	2118.71	1685.48	1685.46
	Std	7.21	7.37	12.13	0.01	10.41	16.81	4.25	0.03	0.02
	Rank	7	4	5	1	9	6	8	3	2
F15	Best	332,373.40	170,995.00	161,041.51	95,661.51	14,209,601.07	252,677.50	7,516,854.07	95,688.99	95,677.95
	Mean	480,825.82	245,627.59	285,726.42	96,109.98	16,696,930.44	367,890.48	7,913,546.51	95,726.60	95,707.22
	Std	74,855.30	49,660.44	77,446.38	1073.22	1,361,809.32	99,043.12	244,376.43	20.58	18.31
	Rank	7	4	5	3	9	6	8	2	1
F16	Best	1613.58	1613.26	1613.04	1612.84	1645.74	1613.29	1646.47	1612.70	1612.97
	Mean	1613.90	1613.82	1613.65	1613.46	1646.83	1613.56	1647.35	1613.14	1613.27
	Std	0.16	0.18	0.26	0.22	0.72	0.13	0.38	0.21	0.12
	Rank	7	6	5	3	8	4	9	1	2
F17	Best	4.27E+08	3.38E+08	3.38E+08	3.25E+08	1.79E+09	3.34E+08	1.48E+09	3.25E+08	3.25E+08
	Mean	5.40E+08	3.99E+08	3.92E+08	3.25E+08	1.90E+09	3.69E+08	1.48E+09	3.25E+08	3.25E+08
	Std	5.42E+07	2.54E+07	4.74E+07	4.47E+02	9.32E+07	2.33E+07	1.59E+07	7.40E+01	6.93E+01
	Rank	7	6	5	3	9	4	8	2	1
F18	Best	1.1436E+10	1.0018E+10	1.0018E+10	1.0016E+10	4.0532E+10	1.0016E+10	3.9518E+10	1.0016E+10	1.0016E+10
	Mean	1.2344E+10	1.1023E+10	1.0752E+10	1.0016E+10	4.2795E+10	1.0418E+10	3.9545E+10	1.0016E+10	1.0016E+10
	Std	4.5746E+08	6.7397E+08	5.8952E+08	2.1281E+01	1.0419E+09	4.6019E+08	3.9749E+07	3.9205E+00	2.2977E+00
	Rank	7	6	5	3	9	4	8	2	1
F19	Best	2479.35	2412.94	2388.13	2379.70	10,973.13	2392.06	10,241.85	2378.28	2378.01
	Mean	2558.96	2495.82	2455.76	2381.06	11,640.88	2428.73	10,276.93	2379.43	2378.89
	Std	43.80	45.65	46.06	1.09	320.71	34.17	33.56	0.60	0.42
	Rank	7	6	5	3	9	4	8	2	1
F20	Best	8.8904E+08	6.9325E+08	6.9322E+08	6.9318E+08	6.6115E+05	6.9321E+08	2.7941E+05	6.9318E+08	6.9318E+08
	Mean	1.2599E+09	8.6846E+08	7.9064E+08	6.9318E+08	8.5743E+05	7.2958E+08	3.1482E+05	6.9318E+08	6.9318E+08
	Std	1.8284E+08	2.2780E+08	2.0671E+08	1.7719E+01	9.0647E+04	9.6233E+07	2.8322E+04	8.0687E+00	3.4602E+00
	Rank	9	8	7	5	2	6	1	4	3
F21	Best	1.3358E+09	7.8918E+08	7.8918E+08	7.8918E+08	4.6016E+08	7.8918E+08	3.8403E+08	7.8918E+08	7.8918E+08
	Mean	1.5792E+09	1.0404E+09	9.3176E+08	7.8918E+08	5.2214E+08	1.0254E+09	3.8548E+08	7.8918E+08	7.8918E+08
	Std	1.3087E+08	1.9006E+08	1.5341E+08	4.0963E+02	3.7660E+07	2.4918E+08	2.7548E+06	1.5085E+02	1.2546E+02
	Rank	9	8	6	5	2	7	1	4	3
F22	Best	1.8761E+06	1.2536E+06	1.2534E+06	1.2528E+06	3.8079E+05	1.2535E+06	3.6752E+05	1.2528E+06	1.2528E+06
	Mean	2.4232E+06	1.5075E+06	1.4324E+06	1.2533E+06	4.6737E+05	1.3894E+06	3.6966E+05	1.2530E+06	1.2530E+06
	Std	3.5813E+05	2.7838E+05	2.9168E+05	2.4260E+02	5.1250E+04	2.2597E+05	1.3882E+03	1.6331E+02	1.2373E+02
	Rank	9	8	7	5	2	6	1	4	3
F23	Best	2500.00	2500.00	2517.34	2500.00	2500.00	2500.00	2500.00	2500.00	2500.00
	Mean	2500.00	2500.00	2545.93	2500.00	2500.00	2500.00	2500.00	2500.01	2500.00
	Std	0.00E+00	0	1.26E+01	2.21E−06	9.13E−13	0	2.07E−13	4.89E−03	2.96E−05
	Rank	1	1	9	6	5	1	4	8	7
F24	Best	2600.00	2600.00	2600.25	2600.00	2600.01	2600.00	2600.00	2601.65	2600.50
	Mean	2600.00	2600.00	2610.15	2602.02	2600.04	2600.00	2600.00	2604.32	2600.81
	Std	1.15E−03	0.00E+00	4.51E+00	1.85E+00	2.15E−02	0.00E+00	4.47E−04	1.97E+00	2.07E−01
	Rank	3	1	9	7	5	1	4	8	6
F25	Best	2700.00	2700.00	2700.38	2700.00	2700.00	2700.00	2700.00	2700.00	2700.00
	Mean	2700.00	2700.00	2700.74	2700.00	2700.00	2700.00	2700.00	2700.00	2700.00
	Std	0.00E+00	0.00E+00	1.59E−01	9.09E−10	1.83E−12	0.00E+00	2.23E−13	2.90E−04	1.47E−06
	Rank	1	1	9	6	5	1	4	8	7
F26	Best	2800.00	2800.00	2800.01	2800.00	2800.00	2800.00	2800.00	2800.00	2800.00
	Mean	2800.00	2800.00	2800.03	2800.00	2800.00	2800.00	2800.00	2800.01	2800.01
	Std	0.00E+00	0.00E+00	1.44E−02	7.66E−12	1.30E−08	2.07E−13	6.00E−05	4.25E−03	3.46E−03
	Rank	1	1	9	4	5	3	6	8	7
F27	Best	2900.00	2900.00	2959.45	2900.00	2900.00	2900.00	2900.00	2900.00	2900.00
	Mean	2900.00	2900.00	3975.98	2900.00	2900.00	2900.00	2900.13	3021.24	2963.95
	Std	0.00E+00	0.00E+00	1.63E+03	2.99E−09	7.83E−13	0.00E+00	7.31E−01	1.94E+02	4.19E+01
	Rank	1	1	9	5	4	1	6	8	7
F28	Best	3000.00	3000.00	3069.40	3000.00	3000.00	3000.00	3000.00	3000.00	3000.00
	Mean	3000.00	3000.00	4567.59	3000.00	3000.00	3000.00	3000.00	3209.08	3149.76
	Std	0.00E+00	0.00E+00	2.23E+03	2.92E−09	2.02E−12	0.00E+00	2.57E−05	2.81E+02	8.02E+01
	Rank	1	1	9	5	4	1	6	8	7
F29	Best	3.10E+03	3100	9,706,580.724	3100.00	3100	3100	3100	4177.03	3125.08
	Mean	3.10E+03	3100	56,600,545.86	3100.00	3100	3100	3100	10,297.95	3201.08
	Std	0.00E+00	0	15,192,070.38	5.21E−03	1.37E−07	0	3.38E−13	5467.43	80.62
	Rank	1	1	9	6	5	1	4	8	7
F30	Best	3.20E+03	3200	2,064,310.154	3200	3200.000583	3200	3200	3360.02	3200.82
	Mean	3.20E+03	3200	3,813,585.33	3200.00	3200.001049	3200.00	3200	3858.60	3205.79
	Std	0.00E+00	0	851,270.6626	0.00	0.000287461	0.00E+00	9.03E−12	434.30	5.03
	Rank	1	1	9	5	6	1	4	8	7
Mean rank		5.43	4.90	6.03	3.57	6.80	4.17	6.13	4.00	3.30
Result		6	5	7	2	9	4	8	3	1
+/=/−		8/2/20	8/0/22	0/2/28	13/2/15	12/0/18	8/2/20	13/0/17	3/7/20	−/−/−/

**Table 3:** The results of each algorithm based on the CEC2017 test function set.

F	Index	Algorithm								
		AOA	COA	CSA	COOT	GJO	RSO	SO	CPA	UCDCPA
F1	Best	6.96E+10	6.40E+10	5.86E+10	5.59E+10	2.33E+11	6.34E+10	1.91E+11	5.59E+10	5.59E+10
	Mean	7.32E+10	7.00E+10	6.70E+10	5.59E+10	2.41E+11	6.60E+10	1.93E+11	5.59E+10	5.59E+10
	Std	1.88E+09	3.17E+09	3.41E+09	2.67E+07	4.20E+09	1.44E+09	1.08E+09	1.88E+07	4.78E+06
	Rank	7	6	5	1	9	4	8	3	2
F3	Best	85,044.97	72,332.55	68,149.83	60,867.5	311,129.87	74,233.75	304,642.3	64,533.78	64,517.17
	Mean	90,479.28	85,647.27	81,644.45	64,245.49	332,312.2	80,393.4	327,971.08	68,794.97	68,164.19
	Std	2847.68	4355.04	6275.45	1737.85	6184.6	2943.46	9416.48	1966.07	1509.82
	Rank	7	6	5	1	9	4	8	3	2
F4	Best	26,488.83	22,108.1	20,314.41	19,393.32	99,000.51	22,653.84	80,416.02	19,393.97	19,393.72
	Mean	28,358.87	23,957.51	23,833.92	19,393.92	103,966.78	25,730.38	81,616.57	19,396.35	19,394.45
	Std	1005.02	723.92	1715.46	1.09	2432.27	2241.11	763.89	3.25	0.49
	Rank	7	5	4	1	9	6	8	3	2
F5	Best	946.24	916.64	838.25	762.31	1707.55	838.28	1410.9	768.81	768.19
	Mean	980.14	966.36	921.17	802.49	1791.65	911.64	1475.34	795.23	791.25
	Std	17.53	18.19	44.59	16.65	43.84	27.96	25.48	10.92	10.18
	Rank	7	6	5	3	9	4	8	2	1
F6	Best	688.63	691.21	672.97	662.55	682.94	676.08	663.69	660.02	657.37
	Mean	701.71	702.21	684.78	667.72	689.74	693.4	666.59	662.5	660.53
	Std	5.81	5.22	7.57	3.06	5.38	6.49	1.48	1.76	1.65
	Rank	8	9	5	4	6	7	3	2	1
F7	Best	1428.41	1361.23	1214.3	1132.17	3236	1336.76	2690.8	1122.69	1112.87
	Mean	1463	1455.69	1374.18	1207.06	3437.53	1391.88	2792.63	1163.45	1137.49
	Std	18.12	36.49	59.98	42.37	78.32	26.1	54.53	23.89	15.34
	Rank	7	6	4	3	9	5	8	2	1
F8	Best	1121.69	1144.29	1020.03	981.54	2135.87	1065.63	1920.28	972.56	970.28
	Mean	1172.48	1176.61	1090.92	1003.63	2282.34	1134	1962.08	992.03	984.31
	Std	20.78	15.85	40.25	13.72	46.93	22.89	26.01	9.41	7.43
	Rank	6	7	4	3	9	5	8	2	1
F9	Best	8120.94	10,311.72	6153.63	4882.99	37,198.65	7647.01	55,433.95	4809.7	9504.28
	Mean	11,063.65	12,331.8	10,051.96	6365.98	65,082.02	10,760.46	71,394.03	6248.02	10,566.12
	Std	1370.08	932.13	2721.43	901.49	18,797.13	1286.47	7660.7	2372.38	541.29
	Rank	6	7	3	2	8	5	9	1	4
F10	Best	8210.32	8449.16	6531.75	4721.36	23,052.26	7194.6	17,350.85	4437.85	4524.28
	Mean	9075.77	9531.32	7835.56	5439.08	24,892.66	8074.75	18,536.66	5101.85	5046.05
	Std	395.89	418.53	948.21	398.42	899.62	533.48	635.83	319	224.01
	Rank	6	7	4	3	9	5	8	2	1
F11	Best	2.16E+07	1.83E+07	1.83E+07	1.83E+07	1.04E+12	1.83E+07	8.79E+11	1.83E+07	1.83E+07
	Mean	6.94E+07	2.02E+07	2.30E+07	1.83E+07	1.59E+12	1.93E+07	8.82E+11	1.83E+07	1.83E+07
	Std	3.13E+07	6.87E+06	1.18E+07	17.18	3.94E+11	2,995,138.49	9,003,578,632	6.05	5.19
	Rank	7	5	6	3	9	4	8	2	1
F12	Best	2.24E+10	2.06E+10	1.91E+10	1.85E+10	1.92E+11	2.02E+10	1.64E+11	1.85E+10	1.85E+10
	Mean	2.49E+10	2.24E+10	2.17E+10	1.85E+10	2.00E+11	2.25E+10	1.65E+11	1.85E+10	1.85E+10
	Std	9.48E+08	9.79E+08	1.25E+09	3.23E+06	3.50E+09	1.49E+09	8.12E+08	5.35E+04	1.98E+04
	Rank	7	5	4	3	9	6	8	2	1
F13	Best	3.30E+10	2.86E+10	2.86E+10	2.86E+10	4.66E+10	2.86E+10	4.41E+10	2.86E+10	2.86E+10
	Mean	3.61E+10	3.09E+10	3.02E+10	2.86E+10	4.85E+10	3.21E+10	4.42E+10	2.86E+10	2.86E+10
	Std	1.46E+09	1.85E+09	1.80E+09	1.54E+02	1.10E+09	2.79E+09	4.77E+07	4.67E+01	3.47E+01
	Rank	7	5	4	3	9	6	8	2	1
F14	Best	3.95E+08	2.86E+08	2.86E+08	2.86E+08	2.08E+08	2.86E+08	1.89E+08	2.86E+08	2.86E+08
	Mean	5.30E+08	3.41E+08	3.23E+08	2.86E+08	2.69E+08	2.98E+08	1.89E+08	2.86E+08	2.86E+08
	Std	8.81E+07	5.86E+07	6.89E+07	1.51E+01	3.70E+07	2.71E+07	1.52E+05	6.54E+00	4.37E+00
	Rank	9	8	7	5	2	6	1	4	3
F15	Best	3.14E+09	2.97E+09	2.97E+09	2.97E+09	2.80E+10	2.97E+09	2.79E+10	2.97E+09	2.97E+09
	Mean	3.66E+09	3.16E+09	3.27E+09	2.97E+09	2.93E+10	3.03E+09	2.79E+10	2.97E+09	2.97E+09
	Std	3.13E+08	1.86E+08	3.88E+08	1.73E+03	7.98E+08	1.21E+08	1.34E+07	8.94E+00	8.55E+00
	Rank	7	5	6	3	9	4	8	2	1
F16	Best	17,902.11	15,436.27	15,509.67	14,481.07	23,217.34	15,864.76	19,668.88	14,458.22	14,473.92
	Mean	20,667.79	17,722.05	17,581.48	15,076.45	25,378.02	16,751.56	20,397.27	14,891.61	14,776.26
	Std	1149.15	1028.18	1436.1	341.67	809.46	555.94	421.89	219.71	166.64
	Rank	8	6	5	3	9	4	7	2	1
F17	Best	66,689.36	57,907.37	57,117.16	56,609.44	26,517,855.16	57,391.92	24,582,274.02	56,590.26	56,589.9
	Mean	102,835.52	61,986.7	62,600.6	56,895.25	32,596,475.94	65,765.57	24,615,145.06	56,771.83	56,736.15
	Std	14,040.69	4745.2	9976.28	172.09	4,126,332.61	12,805.85	163,000.24	107.86	97.93
	Rank	7	4	5	3	9	6	8	2	1
F18	Best	1.97E+09	1.38E+09	1.38E+09	1.37E+09	1.82E+08	1.38E+09	1.61E+08	1.37E+09	1.37E+09
	Mean	2.50E+09	1.67E+09	1.76E+09	1.37E+09	2.33E+08	1.49E+09	1.61E+08	1.37E+09	1.37E+09
	Std	2.69E+08	2.68E+08	4.13E+08	9.87E+05	3.58E+07	1.59E+08	5.02E+05	2.11E+01	2.96E+00
	Rank	9	7	8	5	2	6	1	4	3
F19	Best	3.38E+09	3.12E+09	3.12E+09	3.12E+09	2.86E+10	3.12E+09	2.85E+10	3.12E+09	3.12E+09
	Mean	3.71E+09	3.15E+09	3.26E+09	3.12E+09	2.98E+10	3.35E+09	2.85E+10	3.12E+09	3.12E+09
	Std	2.72E+08	3.86E+07	1.95E+08	3.13E+03	8.12E+08	2.85E+08	8.67E+05	5.05E+00	5.14E+00
	Rank	7	4	5	3	9	6	8	2	1
F20	Best	3.45E+03	3.42E+03	3.32E+03	3.22E+03	6.29E+03	3.36E+03	5.50E+03	3.22E+03	3.21E+03
	Mean	3.73E+03	3.65E+03	3.54E+03	3.31E+03	6.98E+03	3.47E+03	6.08E+03	3.27E+03	3.26E+03
	Std	1.25E+02	8.94E+01	1.21E+02	7.20E+01	4.18E+02	1.09E+02	2.79E+02	3.32E+01	3.81E+01
	Rank	7	6	5	3	9	4	8	2	1
F21	Best	2.97E+03	2.91E+03	2.82E+03	2.73E+03	7.69E+03	2.89E+03	6.04E+03	2.74E+03	2.74E+03
	Mean	3.02E+03	2.96E+03	2.92E+03	2.78E+03	7.97E+03	2.93E+03	6.15E+03	2.75E+03	2.75E+03
	Std	2.89E+01	2.57E+01	5.49E+01	2.67E+01	1.86E+02	2.75E+01	6.98E+01	1.51E+01	7.63E+00
	Rank	7	6	4	3	9	5	8	2	1
F22	Best	9.94E+03	9.91E+03	8.62E+03	7.61E+03	2.74E+04	9.24E+03	2.21E+04	7.29E+03	7.31E+03
	Mean	1.06E+04	1.06E+04	9.61E+03	8.15E+03	2.90E+04	9.94E+03	2.32E+04	7.64E+03	7.57E+03
	Std	3.33E+02	3.34E+02	7.18E+02	3.23E+02	9.22E+02	3.44E+02	4.74E+02	2.02E+02	1.82E+02
	Rank	7	6	4	3	9	5	8	2	1
F23	Best	5.70E+03	5.78E+03	5.18E+03	4.58E+03	1.06E+04	5.54E+03	8.46E+03	4.45E+03	4.37E+03
	Mean	6.39E+03	6.22E+03	5.81E+03	4.88E+03	1.12E+04	5.88E+03	8.99E+03	4.63E+03	4.57E+03
	Std	2.51E+02	1.79E+02	3.94E+02	1.96E+02	3.53E+02	1.97E+02	1.89E+02	8.54E+01	8.39E+01
	Rank	7	6	4	3	9	5	8	2	1
F24	Best	4.98E+03	4.85E+03	4.80E+03	4.64E+03	1.46E+04	4.85E+03	1.31E+04	4.64E+03	4.64E+03
	Mean	5.02E+03	4.98E+03	4.91E+03	4.65E+03	1.48E+04	4.93E+03	1.31E+04	4.66E+03	4.65E+03
	Std	2.50E+01	4.77E+01	5.59E+01	1.76E+01	1.12E+02	4.68E+01	5.86E+01	1.67E+01	1.89E+01
	Rank	7	6	4	1	9	5	8	3	2
F25	Best	6.74E+03	5.78E+03	5.48E+03	5.09E+03	2.45E+04	5.88E+03	2.05E+04	5.09E+03	5.09E+03
	Mean	7.30E+03	6.35E+03	6.25E+03	5.09E+03	2.56E+04	6.42E+03	2.07E+04	5.09E+03	5.09E+03
	Std	2.62E+02	4.38E+02	4.59E+02	2.67E+00	5.73E+02	4.09E+02	1.04E+02	4.11E−01	3.13E−01
	Rank	7	5	4	3	9	6	8	2	1
F26	Best	1.35E+04	1.28E+04	1.21E+04	1.13E+04	5.02E+04	1.25E+04	4.33E+04	1.13E+04	1.12E+04
	Mean	1.40E+04	1.36E+04	1.31E+04	1.15E+04	5.20E+04	1.30E+04	4.39E+04	1.14E+04	1.14E+04
	Std	2.91E+02	3.24E+02	5.53E+02	1.35E+02	7.89E+02	3.38E+02	4.56E+02	1.02E+02	7.71E+01
	Rank	7	6	5	3	9	4	8	2	1
F27	Best	8.04E+03	8.25E+03	6.84E+03	6.31E+03	1.90E+04	7.64E+03	1.63E+04	6.29E+03	6.15E+03
	Mean	8.74E+03	8.71E+03	7.86E+03	6.71E+03	2.00E+04	8.08E+03	1.69E+04	6.52E+03	6.47E+03
	Std	2.87E+02	2.00E+02	5.73E+02	2.20E+02	4.24E+02	1.84E+02	3.19E+02	1.36E+02	1.80E+02
	Rank	7	6	4	3	9	5	8	2	1
F28	Best	8.87E+03	7.97E+03	7.94E+03	7.19E+03	3.33E+04	8.02E+03	2.87E+04	7.19E+03	7.19E+03
	Mean	9.15E+03	8.60E+03	8.52E+03	7.19E+03	3.43E+04	8.51E+03	2.91E+04	7.20E+03	7.19E+03
	Std	1.70E+02	2.23E+02	3.24E+02	1.12E+00	5.45E+02	2.89E+02	1.87E+02	1.06E+00	7.13E−01
	Rank	7	6	5	1	9	4	8	3	2
F29	Best	5.87E+04	4.59E+04	3.51E+04	3.15E+04	1.08E+06	3.67E+04	7.26E+05	3.15E+04	3.15E+04
	Mean	7.46E+04	5.64E+04	4.70E+04	3.19E+04	1.22E+06	4.58E+04	7.62E+05	3.18E+04	3.16E+04
	Std	9.69E+03	5.89E+03	9.72E+03	4.84E+02	1.12E+05	6.61E+03	2.34E+04	3.38E+02	2.46E+02
	Rank	7	6	5	3	9	4	8	2	1
F30	Best	7.38E+09	5.97E+09	6.16E+09	5.65E+09	4.42E+10	6.19E+09	3.67E+10	5.65E+09	5.65E+09
	Mean	8.15E+09	6.96E+09	6.77E+09	5.65E+09	4.56E+10	6.64E+09	3.73E+10	5.65E+09	5.65E+09
	Std	3.11E+08	4.41E+08	3.96E+08	1.54E+06	7.63E+08	5.17E+08	4.18E+08	1.85E+06	4.04E+05
	Rank	7	6	5	1	9	4	8	3	2
Mean rank		7.1	5.97	4.76	2.72	8.38	4.97	7.34	2.31	1.45
Result		7	6	4	3	9	5	8	2	1
+/=/−		2000/1/28	0/0/29	2000/1/28	2008/1/20	2/0/27	2000/1/28	2/0/27	1/13/15	−/−/−/

**Table 4:** Results for the comparison of algorithm performance in the pressure vessel design issue.

Algorithms	*T_s_*	*T_h_*	*R*	*L*	Optimum Cost
BWO [42]	1.35762647	1.093437138	67.69082252	113.0682817	7452.833749
GSA [16]	0.90125717	0.881298174	46.32687683	167.7379974	8900.046643
AOA [43]	0.780590299	0.385783281	40.44361782	198.3877341	5894.187289
AO [44]	0.847809404	0.433226318	43.66107194	160.3307465	6149.803509
HHO [28]	0.882129626	0.438803464	45.70354005	136.5566478	6098.410688
RSO [39]	1.023595723	0.548121786	53.00397707	78.53244426	6752.320707
SCA [25]	0.828990697	0.453075845	42.86366246	171.8211562	6238.443072
WOA [26]	0.845217747	0.414401057	42.25129909	174.7516586	6193.691409
PSO [23]	0.789311349	0.389923651	40.87077061	192.4817157	5907.740366
SHO [45]	1.45363783	0.864019419	69.21011101	10	10,953.58287
MVO [13]	0.83997938	0.41617848	43.49393648	160.2903645	6011.610548
HS [19]	1.057860805	0.616265357	53.79451517	128.7466594	9381.627416
CPA [29]	0.779207674	0.385163555	40.37358135	199.2501794	5887.103014
UCDCPA	0.818807403	0.404738252	42.42550141	172.6257773	5885.317546

**Table 5:** Statistical results for the algorithms in the pressure vessel design problem.

Algorithms	Best	Mean	Worst	Std
BWO [42]	7452.833749	8668.096187	9835.931831	645.6007505
GSA [16]	8900.046643	22,826.83618	33,709.70179	6739.746909
AOA [43]	5894.187289	6073.39365	6467.034139	176.6077507
AO [44]	6149.803509	6703.38539	7650.836881	445.6648705
HHO [28]	6098.410688	6773.338116	7321.578979	373.4757438
RSO [39]	6752.320707	13,303.19277	36,017.19213	7139.553858
SCA [25]	6238.443072	6740.990306	8667.698978	596.9230292
WOA [26]	6193.691409	7788.515478	14,356.43308	2207.831263
PSO [23]	5907.740366	6215.186207	6817.609346	308.9590064
SHO [45]	10,953.58287	20,683.71124	38,772.72675	7522.154569
MVO [13]	6011.610548	6454.018394	7244.10122	305.5658618
HS [19]	9381.627416	13,219.79192	16,750.36369	2431.426944
CPA [29]	5887.103014	6011.158017	6307.525495	1.25E+02
UCDCPA	5885.317546	5885.317546	5885.317546	1.87E−12

**Table 6:** Results for the comparison of algorithm performance in the welded beam design issue.

Algorithms	*h*	*l*	*t*	*b*	Optimum Cost
GSA [16]	0.162851394	4.54597146	8.638236427	0.250005147	2.060084936
AOA [43]	0.204277902	3.303984088	9.04125477	0.205706549	1.700621531
AO [44]	0.189374632	3.547522386	9.134755541	0.206786696	1.73521502
HHO [28]	0.188109377	3.905038631	9.030136455	0.206025348	1.755250851
RSO [39]	0.149913681	6.263663498	8.735493845	0.223742119	2.060920727
SCA [25]	0.197001179	3.447630359	9.061850711	0.210734829	1.750779615
WOA [26]	0.187626514	3.496137108	9.55526453	0.203278952	1.770945189
GWO [24]	0.205639834	3.258414504	9.038918564	0.205727445	1.696210777
PSO [23]	0.205717244	3.253567261	9.036950989	0.205728009	1.695333367
SHO [45]	0.15716373	4.788258496	9.265491874	0.243779909	2.172339171
HS [19]	0.133846407	5.896646319	9.090787017	0.239299182	2.199069081
CPA [29]	0.20572964	3.253120041	9.03662391	0.20572964	1.695247165
UCDCPA	0.20572964	3.253120041	9.03662391	0.20572964	1.695247165

**Table 7:** Statistical outcomes for the algorithms in the welded beam design problem.

Algorithms	Best	Mean	Worst	Std
GSA [16]	2.060084936	2.260378901	2.395427434	0.103945911
AOA [43]	1.700621531	2.010126216	3.41715675	0.456539458
AO [44]	1.73521502	1.858312119	2.045979674	0.089512879
HHO [28]	1.755250851	1.843976042	2.196985884	0.105249207
RSO [39]	2.060920727	5.242901347	46.8049853	9.818645738
SCA [25]	1.750779615	1.83018928	1.927682829	0.043302748
WOA [26]	1.770945189	2.078361923	3.598644235	0.456109982
GWO [24]	1.696210777	1.698075685	1.706826075	0.002303005
PSO [23]	1.695333367	1.696054342	1.700382568	0.001099938
SHO [45]	2.172339171	8.41388697	57.61069741	12.82400304
HS [19]	2.199069081	2.961271545	3.338760975	0.264197415
CPA [29]	1.695247165	1.695247165	1.695247165	1.14E−16
UCDCPA	1.695247165	1.695247165	1.695247165	0

**Table 8:** Results for the comparison of algorithm performance in the TCSD problem.

Algorithms	*d*	*D*	*N*	Optimum Cost
GSA [16]	0.054336606	0.411182717	9.27940294	0.013693233
AO [44]	0.053858024	0.382210602	11.05851677	0.014477628
HHO [28]	0.055518108	0.456022463	7.191402658	0.012919253
RSO [39]	0.051578783	0.351426469	11.86143225	0.012959396
WOA [26]	0.053192553	0.393977515	9.397755032	0.012705521
PSO [23]	0.052695325	0.379080507	10.26803936	0.012913703
SMA [48]	0.050025201	0.317991335	13.98116224	0.012717488
SHO [45]	0.05	0.314726583	15	0.01337588
MVO [13]	0.05	0.316210131	14.19550214	0.012802955
HS [19]	0.054112945	0.413019501	9.910684619	0.014404879
ALO [49]	0.050987891	0.340082479	12.33528746	0.012674323
CPA [29]	0.051858341	0.360803904	11.05336423	0.012665752
UCDCPA	0.052565382	0.378168224	10.13392929	0.012665231

**Table 9:** Statistical outcomes for the algorithms in the TCSD problem.

Algorithms	Best	Mean	Worst	Std
GSA [16]	0.013693233	0.017446335	0.021225117	0.002099083
AO [44]	0.014477628	0.016524122	0.020944277	0.001749639
HHO [28]	0.012919253	0.01367488	0.015510811	0.000733611
RSO [39]	0.012959396	4176.97903	42208.32165	10463.97555
WOA [26]	0.012705521	0.013497022	0.017773562	0.001085082
PSO [23]	0.012913703	0.013681989	0.014907415	0.000629188
SMA [48]	0.012717488	0.013097227	0.014225745	0.00051897
SHO [45]	0.01337588	1562.290559	16836.22462	4721.617609
MVO [13]	0.012802955	0.01692502	0.018300031	0.001618284
HS [19]	0.014404879	0.023626948	0.050181296	0.007737902
ALO [49]	0.012674323	0.013843033	0.017642955	0.001814955
CPA [29]	0.012665752	0.012762078	0.013068728	0.00012129
UCDCPA	0.012665231	0.012665231	0.012665232	2.66E−10

**Table 10:** Comparison of algorithm performance for the best designs in the gear design problem.

Algorithms	*x*_1_	*x*_2_	*x*_3_	*x*_4_	Optimum Cost
GSA [16]	49.09926771	16.58870178	19.96015692	43.40356866	2.700857E−12
AOA [43]	43.71647858	19.7208177	16.4232225	49.45682569	2.700857E−12
AO [44]	49.83084862	16.24845435	19.71874271	43.324778	2.700857E−12
RSO [39]	27.29887237	12	12	37.17778148	1.827380E−08
SCA [25]	49.86378029	19.81902942	16.50739713	43.29977556	2.700857E−12
GWO [24]	49.92736013	19.55850161	16.4773682	43.05012785	2.700857E−12
PSO [23]	49.33432112	19.39566196	16.54612986	43.10005969	2.700857E−12
SMA [48]	51.70131506	13.74783833	30.30206275	53.45404325	2.307816E−11
SHO [45]	55.97209247	40.47794844	12	60	1.381144E−06
WHO [51]	43.86923887	19.45848526	16.13971724	49.58874223	2.700857E−12
MVO [13]	43.18211959	19.81774931	16.368027	49.42425551	2.700857E−12
MFO [27]	51.02233936	26.4068411	15.37123392	53.66313366	2.307816E−11
ALO [49]	53.90915117	20.30000998	13.44122459	34.0496304	2.307816E−11
CPA [29]	43.9192177	19.83148672	16.99034087	49.37036408	2.700857E−12
UCDCPA	49.97261094	19.91168483	16.87627009	43.89722598	2.700857E−12

**Table 11:** Statistical outcomes for the algorithms in the gear train design problem.

Algorithms	Best	Mean	Worst	Std
GSA [16]	2.700857E−12	1.010642E−09	2.357641E−09	9.394177E−10
AOA [43]	2.700857E−12	2.442156E−09	1.827380E−08	4.358741E−09
AO [44]	2.700857E−12	1.248539E−09	4.503304E−09	1.289949E−09
RSO [39]	1.827380E−08	1.542447E−04	1.646172E−03	3.713617E−04
SCA [25]	2.700857E−12	9.134559E−10	2.357641E−09	7.666596E−10
GWO [24]	2.700857E−12	1.138729E−10	9.921580E−10	3.005126E−10
PSO [23]	2.700857E−12	6.440009E−11	9.921580E−10	2.185927E−10
SMA [48]	2.307816E−11	4.989225E−09	2.726451E−08	7.559668E−09
SHO [45]	1.381144E−06	1.471356E−04	8.349042E−04	2.498206E−04
WHO [51]	2.700857E−12	2.655999E−10	9.921580E−10	3.816328E−10
MVO [13]	2.700857E−12	4.861756E−10	2.357641E−09	6.039829E−10
MFO [27]	2.307816E−11	1.850996E−09	4.503304E−09	1.318984E−09
ALO [49]	2.307816E−11	3.192183E−09	1.827380E−08	4.885282E−09
CPA [29]	2.700857E−12	4.639657E−10	4.503304E−09	1.112919E−09
UCDCPA	2.700857E−12	1.858504E−11	1.166116E−10	2.521680E−11

**Table 12:** Comparison of algorithm performances for the best designs in the cantilever beam problem.

Algorithms	*x*_1_	*x*_2_	*x*_3_	*x*_4_	*x*_5_	Optimum Cost
BWO [42]	1.91112381	3.371424243	3.136849543	3.984923889	1.830067654	0.895341162
GSA [16]	2.742172682	2.227520474	2.039099093	1.600194337	0.987632636	0.685438760
AOA [43]	2.837698938	1.949856649	1.857736678	1.523619661	1.031383307	0.697298770
AO [44]	2.726339704	2.243634883	2.035880163	1.580650671	0.97798304	0.685429422
RSO [39]	2.397288674	2.002288903	1.594785803	2.701129185	0.513855729	0.941856920
SCA [25]	2.7851993	2.163499301	1.945893374	1.639149628	1.082000068	0.687731007
PSO [23]	3.336873677	2.237796936	1.603377869	1.935560703	0.831736446	0.726407620
SHO [45]	2.782322365	2.254589461	2.42138236	1.930452462	1.056199247	0.704087739
HS [19]	3.094335154	1.649156667	7.852158097	1.758757853	1.270419628	1.051561683
CPA [29]	2.732785709	2.228074467	2.046348837	1.590249628	0.977632633	0.685408058
UCDCPA	2.732172679	2.228520482	2.041099089	1.590194328	0.977632631	0.685406037

**Table 13:** Statistical results for the algorithms in the cantilever beam design problem.

Algorithms	Best	Mean	Worst	Std
BWO [42]	0.895341162	0.989495691	1.095570621	0.057368242
GSA [16]	0.685438760	0.685424155	0.685768406	8.10282E−05
AOA [43]	0.697298770	0.743549727	0.813673683	0.027671128
AO [44]	0.685429422	0.685533512	0.685860485	0.000101308
RSO [39]	0.941856920	2.016025356	4.559510955	1.097470827
SCA [25]	0.687731007	0.693337949	0.707001376	0.00448995
PSO [23]	0.726407620	0.816503624	0.914341693	0.048147663
SHO [45]	0.704087739	2.0610002	5.193706033	1.737868709
HS [19]	1.051561683	1.529689345	2.035159822	0.287530949
CPA [29]	0.685408058	0.685408058	0.685408058	1.13875E−16
UCDCPA	0.685406037	0.685406037	0.685406037	1.13906E−16

## Data Availability

All data generated or analyzed during this study were included in this published article.
